# Impact of aging on treatment considerations for multiple sclerosis patients

**DOI:** 10.3389/fneur.2023.1197212

**Published:** 2023-07-07

**Authors:** Gabrielle Macaron, Catherine Larochelle, Nathalie Arbour, Manon Galmard, Jean Marc Girard, Alexandre Prat, Pierre Duquette

**Affiliations:** ^1^Centre Hospitalier de l’Université de Montréal, Montréal, QC, Canada; ^2^Department of Neurosciences, Faculty of Medicine, Université de Montréal, Montréal, QC, Canada; ^3^Neuroimmunology Research Laboratory, Centre de Recherche du Centre Hospitalier de l’Université de Montréal, Montréal, QC, Canada; ^4^Faculté de Médecine, Université Saint-Joseph de Beyrouth, Beirut, Lebanon

**Keywords:** multiple sclerosis (MS), aging, comorbidity, treatment efficacy and safety, treatment discontinuation

## Abstract

With a rapidly aging global population and improvement of outcomes with newer multiple sclerosis (MS)-specific disease-modifying therapies (DMTs), the epidemiology of MS has shifted to an older than previously described population, with a peak prevalence of the disease seen in the 55–65 years age group. Changes in the pathophysiology of MS appear to be age-dependent. Several studies have identified a consistent phase of disability worsening around the fifth decade of life. The latter appears to be independent of prior disease duration and inflammatory activity and concomitant to pathological changes from acute focal active demyelination to chronic smoldering plaques, slow-expanding lesions, and compartmentalized inflammation within the central nervous system (CNS). On the other hand, decreased CNS tissue reserve and poorer remyelinating capacity with aging lead to loss of relapse recovery potential. Aging with MS may imply longer exposure to DMTs, although treatment efficacy in patients >55 years has not been evaluated in pivotal randomized controlled trials and appears to decrease with age. Older individuals are more prone to adverse effects of DMTs, an important aspect of treatment individualization. Aging with MS also implies a higher global burden of comorbid illnesses that contribute to overall impairments and represent a crucial confounder in interpreting clinical worsening. Discontinuation of DMTs after age 55, when no evidence of clinical or radiological activity is detected, is currently under the spotlight. In this review, we will discuss the impact of aging on MS pathobiology, the effect of comorbidities and other confounders on clinical worsening, and focus on current therapeutic considerations in this age group.

## Introduction

The field of multiple sclerosis (MS) has grown considerably in the past 25 years, from the refinement of the diagnostic criteria to the expansion of the therapeutic arsenal. We now have a better understanding of the pathophysiological mechanisms driving clinical worsening and early factors impacting prognosis, such as baseline disease characteristics and treatment effect modifiers. Knowledge on the clinical potential of biomarkers of disease severity, prognosis, and treatment response has also significantly increased. Most of these advances are most useful for the early phases of MS; however, advances in the field of late-stage and progressive MS remain modest. With a rapidly aging global population and improvement of outcomes with newer MS-specific disease-modifying therapies (DMTs), the epidemiology of MS has shifted to an older than previously described population. According to US-based health claims data, MS prevalence estimates demonstrate an overall aging of the MS population, with the highest prevalence of the disease seen in the 55–65 years age group (1). As an illustrative example, the peak age-specific prevalence in Manitoba, Canada, was seen in patients aged 35–39 years in 1984 vs. 55–59 years in 2004 (2).

Although the disease course is no longer dichotomized into a relapsing–remitting and a progressive course, as there is current consensus that progression independent from relapse activity (PIRA) starts at the earliest stages of the disease (3–5), natural history cohorts suggest that a consistent phase of overt clinical disability worsening is observed in most patients around the fifth decade of life (6–8). Moreover, patients with a primary progressive disease course tend to present at a later age than those with relapsing–remitting disease, and pediatric-onset MS almost exclusively presents with relapses. Importantly, while most DMTs are effective in reducing the acute inflammatory component of the disease – and are hence beneficial in younger patients with early relapsing–remitting MS, therapeutic options for patients with a predominantly progressive course are lacking. Treatment efficacy in patients older than 55 years has not been evaluated in pivotal randomized controlled trials. Nevertheless, an increasing number of large cohort studies have documented decreased DMT efficacy with age (9). In clinical practice, detecting a transition from a predominantly relapsing to a secondary progressive course is often difficult. Clinical disease activity can also be challenging to diagnose in older individuals, as aging is associated with a higher global burden of comorbid illnesses that contribute to overall impairments and represent a crucial confounder in the interpretation of clinical worsening (10, 11). Radiological disease activity may also be difficult to interpret in patients with cardiovascular risk factors who accumulate non-specific microangiopathic lesions on MRI over time. Patients often remain in this “transitional zone” for several years. Because of the lack of clear clinical practice guidelines in this setting (12), DMTs are often continued for decades after the diagnosis. Hence, aging with MS may imply longer exposure to DMTs and cumulative toxicity of sequential DMTs. On the other hand, older individuals are more prone to adverse effects of DMTs (13), an important aspect of treatment individualization. In this review, we discuss the impact of aging on MS pathology, the effect of comorbidities and other confounders on clinical worsening, and we focus on current therapeutic considerations in aging MS patients. We differentiate between late-onset MS (patients with an onset of the disease at an older age) and long-standing MS in aging patients, and will solely focus on the latter.

## The effect of aging on MS pathophysiology

### MS pathophysiology

MS is a sex-biased neuroinflammatory and neurodegenerative disease of the CNS. The hallmarks of MS neuropathology include multifocal areas of demyelination (lesions or plaques), neuroaxonal injury/loss, gliosis, inflammation, and infiltration of peripheral immune cells. Of note, diffuse neuroglial alterations in non-lesional areas, as well as slowly expanding lesions characterized by a rim of activated microglia with iron accumulation at the lesion edge, and subpial demyelination, are considered relatively unique to MS (14–18). These neuropathological characteristics, so far not reported in other CNS demyelinating disorders, are increasingly associated with the distinct course of MS progression occurring independently of relapses (18). Spontaneous remyelination is associated with improved function and reduced disability in MS, but is generally limited, especially in older individuals in the context of chronic inflammation, oxidative injury, and accumulation of debris/injury (5, 19–22).

Pathophysiological mechanisms underlying MS onset implicate the interaction between multiple predisposing genetic risk factors, such as the major histocompatibility complex class I and II (MHC I and II), polymorphisms, and environmental risk factors, such as exposure to EBV, low vitamin D levels, smoking, and obesity (23, 24). Major genetic and environmental risk factors for MS onset directly and indirectly influence the activation and trafficking of immune cells and consequently contribute to the greater risk of MS onset in susceptible individuals (25–27). In line with this, therapeutic approaches targeting peripheral T and B lymphocytes are highly effective, especially in younger people with MS, establishing the crucial contribution of peripheral immune cells to CNS neuroinflammatory processes in MS.

The biological mechanisms underlying the heterogeneous rate and severity of disability accumulation, e.g., the disease course, remain however poorly understood. Few genetic risks loci coding for genes highly expressed in neuroglial cells and linked to cognitive function were recently potentially associated with the severity of MS course, as were genetic polymorphisms associated with educational achievements, a proxy for cognitive reserve (28). In addition to older age being the greatest risk factor for onset of clinically overt progression in MS and for incomplete recovery from relapses (4, 29), this suggests a major contribution of the neurodegenerative aspects of MS to disease course and severity. Considering the increasingly recognized contribution of the immune system to other neurodegenerative disorders, as well as the modest but significant impact of a subset of DMTs on the progressive phase of the disease, neurodegeneration in MS is likely fueled by immune and CNS processes shaped by age-related alterations that tip the balance between immune-mediated injury and repair (5).

### Biological aging

Biological aging is characterized by functional decline and loss of homeostasis over time. The combined accumulation of damage and exhaustion of repair/compensatory mechanisms partake in biological aging. Hallmarks of aging such as the accumulation of nuclear and mitochondrial DNA mutations, telomere attrition, epigenetic alterations, loss of proteostasis, disabled macroautophagy, dysregulated nutrient sensing, mitochondrial dysfunction, cellular senescence, stem cell exhaustion, altered intercellular communication, and gut dysbiosis, contribute to generate the state of chronic low-grade inflammation referred to as inflammaging (30).

#### Aging alters the innate and the adaptive immune systems

**Figure 1:**
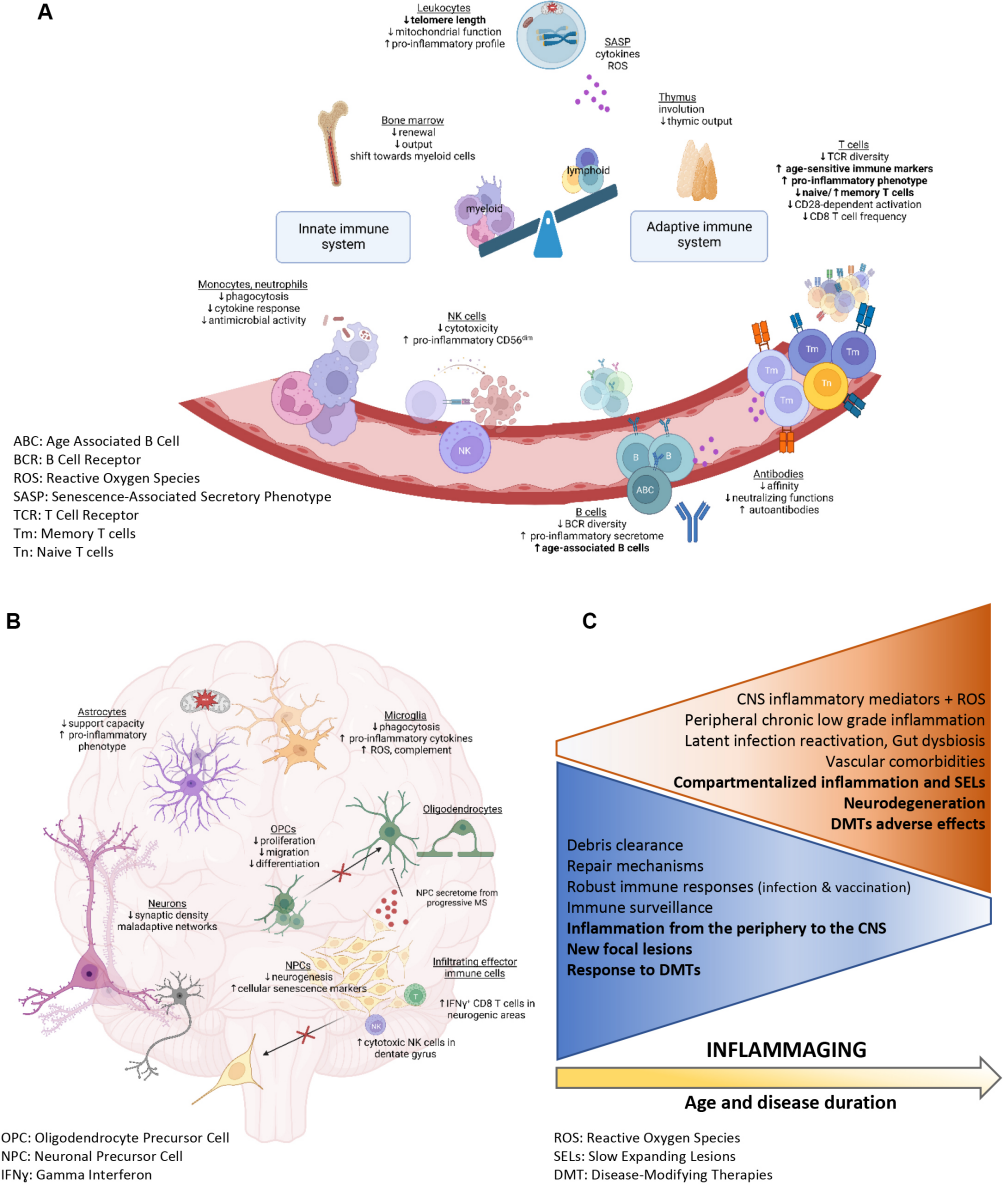
Peripheral immune system and CNS alterations associated with aging (**A,B** created with BioRender.com). **(A–C)** Summary of the physiological functions and characteristics that are altered with age. **(A)** Immunosenescence in the periphery. Alterations reported as enhanced in patients with MS compared with age-matched controls are identified in bold. **(B)** Physiological mechanisms altered in the aging CNS and potentially relevant to MS pathobiology. **(C)** Physiological functions decreasing (blue triangle) or increasing (orange triangle) with age and associated with inflammaging (in bold: MS-specific changes).

As put into light during the SARS-CoV-2 pandemic (31–34), biological aging of the immune system leads to a progressive deterioration of the capacity to mount an appropriate robust immune response, reduced immune surveillance, autoimmunity, and excessive levels of pro-inflammatory mediators. Immunosenescence affects the innate and adaptive immunity (35, 36), with a pronounced impact on lymphocytes and on CNS resident immune cells, the microglia (Figures 1A-C) (37–39). Aging is associated with an altered immune cell output from the bone marrow. The volume of hematopoietic tissue within the bone marrow decreases as individuals age, and bone marrow hematopoietic stem cells exhibit a decreased capacity to self-renew and a shift toward myeloid cell differentiation (40, 41). The thymus involution starts as early as adolescence and data from animal models suggest that male sex hormones accelerate this process (42). Therefore, with aging, more neutrophils and monocytes but less T and B lymphocytes are generated (43). Despite the increased numbers of myeloid cells with aging, defective innate functions are observed. The antimicrobial functions, cytokine responses, and phagocytosis capacity of neutrophils and monocytes are diminished (44). NK cells are also more frequent in older individuals but show reduced cytotoxicity and proliferation and an enriched pro-inflammatory CD56^dim^ phenotype (45). Similarly, aging dampens the capacity of microglia to clear debris and phagocyte proteins (46) but increases their production of pro-inflammatory cytokines, complement and reactive oxygen species (ROS) (47, 48).

The reduced renewal of T and B lymphocytes is partially compensated by homeostatic proliferation, leading to a decreased diversity of the T-cell and B-cell receptor (TCR and BCR) repertoires upon aging. A recent publication revealed a more severe TCR diversity loss in CD8 than CD4 T cells with aging (49). The reduced TCR repertoire diversity is associated with a diminished capacity to mount an efficient cellular immune response targeting encountered pathogens, such as JC virus (50), or tumorigenic cells (51). Increased proportions of dysfunctional regulatory lymphocytes and of terminally differentiated pro-inflammatory T cells are observed upon aging (29). CD4 T lymphocytes from older subjects show impaired autophagy and mitochondrial dysfunction leading to a Th17 profile (52). Moreover, highly differentiated T lymphocytes develop a pro-inflammatory phenotype reminiscent of the senescence associated secretory phenotype (SASP) (53). Aging is furthermore associated with numerous changes in the B cell compartment, including an enhanced proinflammatory B cell secretome or SASP (43). In the elderly, infection or immunization induces antibodies with lower affinity and neutralizing functions, whereas autoantibody levels increase. An age-associated specific subset coined Age-associated B cells (ABCs) has been shown to contribute to the autoantibody secretion (54), notably, ABCs are more prevalent in patients with autoimmune diseases, including MS (55).

#### Immunosenescence fuels neurodegenerative processes

An increasing number of studies suggest that short telomere length in peripheral leukocytes represents a surrogate marker of biological aging, associated with an elevated risk of developing diseases, including neurodegenerative diseases (56, 57). Senescent immune cells cause accelerated systemic aging and are associated with organ damage including in the CNS (48, 58–60). In particular, dysfunctional aged myeloid cells contribute to neurodegenerative processes and age-related cognitive decline in multiple diseases such Alzheimer’s disease, Parkinson’s disease, amyotrophic lateral sclerosis, and MS (61, 62). Exposure to rejuvenating interventions and to young bone marrow-derived immune cells can attenuate the age-related myeloid cell dysfunctions in animal models (63–65). Moreover, NK cells could accumulate in the dentate gyrus upon aging and show cytotoxicity towards neuroblasts, impairing synaptic plasticity and promoting cognitive decline (66). Interestingly, CD8 T lymphocytes showing clonal expansion are found in neurogenic regions of old animals, and their interferon-γ production could interfere with neural stem cell proliferation (Figure 1B) (67). Moreover, Th17 cells, which are increased upon aging, are implicated in a deleterious crosstalk with senescent cells such as fibroblasts (68). Notably, Th17 cells can form prolonged contact with oligodendrocytes in neuroinflammatory conditions and induce loss of distal myelinating processes followed by oligodendrocytic cell death (69).

Multiple age-related mechanisms can reduce the remyelination and neuroregeneration capacity observed in the elderly. Age-related sex hormone deficiencies, e.g., menopause and andropause, contribute to alterations in peripheral and central immune cell response and influence neurodegenerative processes (70). Increased oxidative stress, impaired phagocytosing capacity of myeloid cells (microglia and macrophages) (71), alterations in mitochondrial function and myelin biology, and reduced functionalities (migration, proliferation, differentiation) of oligodendrocyte precursor cells (72) have been identified as culprits in impaired remyelination (Figure 1B). Moreover, decreased neurogenesis, compromised support from astrocytes (73), reduced synaptic density, and maladaptive neuronal network alterations (48) participate in the age-related impaired neurodegeneration. Neural progenitor cells from subjects with progressive MS express markers of cellular senescence *in situ* and *in vitro*, and their secretome induces expression of senescence genes in OPCs and inhibits their differentiation (Figure 1B) (74). Recent studies suggest that aging of neuroglial cells shapes the clinical course and immune response in an animal model of MS (37). Therefore, concomitant age-sensitive processes in the peripheral immune and CNS compartment could contribute to the clinical and immunological shift seen over time in people with MS, from a relapsing to a progressive form. Such coexistent processes parallel a shift from aberrant peripheral immune cell activation and immune cell CNS infiltration to the subsequently intrathecal/diffuse CNS inflammation observed in later phases of MS.

**Figure 2:**
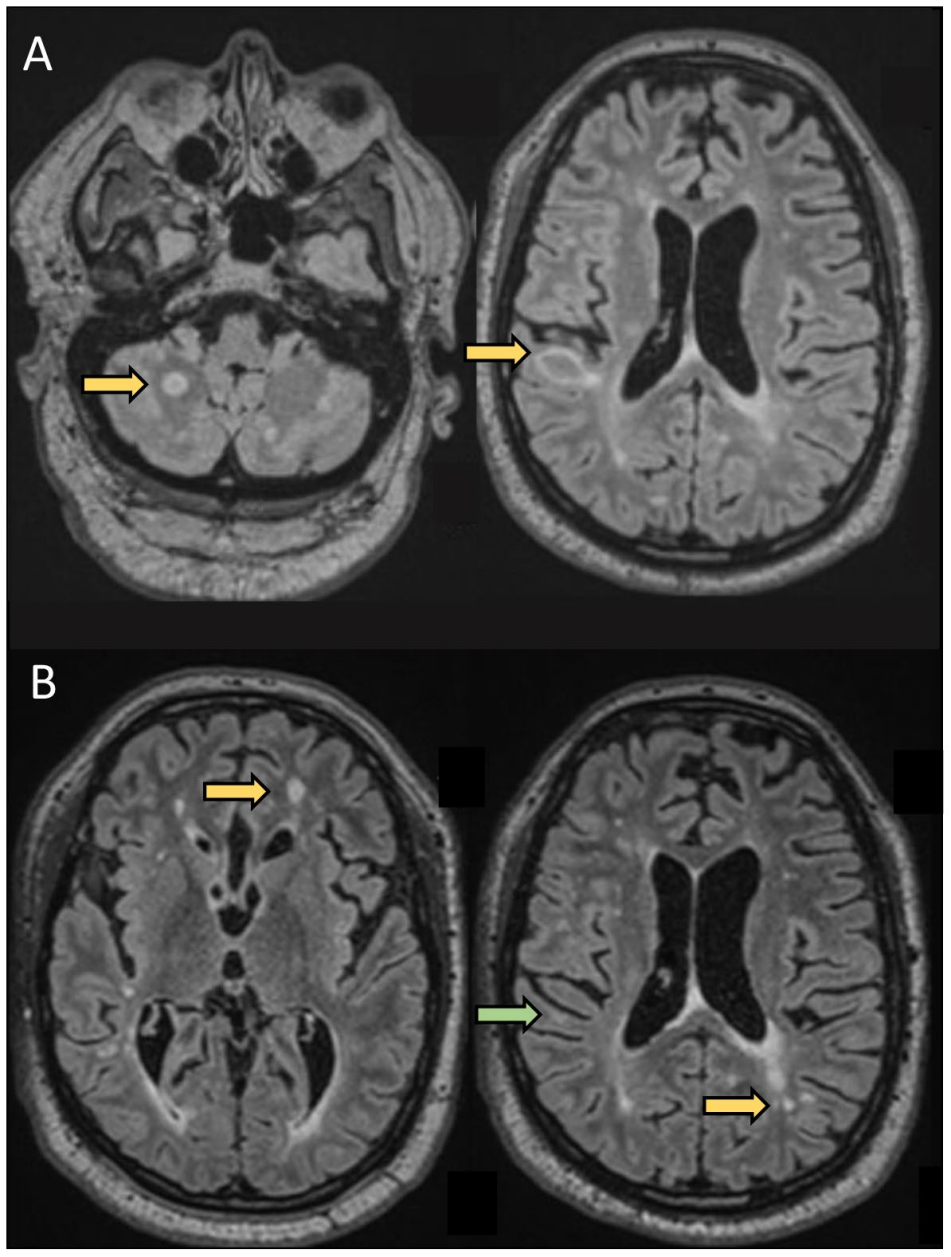
A 61-year-old man was evaluated for new neurological symptoms suggestive of a relapse. He was diagnosed with RRMS in 1998 after two episodes compatible with sub-acute sensory myelitis in 1994 and 1998. He did not receive any treatment for his MS between 1998 and 2019. Between 2000 and 2008, he had a couple of episodes suggestive of mild relapses. He had also noticed some progressively worsening gait instability and cognitive difficulties over the past few years, In April 2019 (at the age of 57), he experienced transient tingling followed by mild weakness and ataxia of the left upper extremity which resolved spontaneously over 5 weeks. Brain MRI showed 2 new lesions, one in the right juxtacortical posterior frontal lobe in the precentral gyrus explaining his symptoms. He was started on dimethyl fumarate 240 mg BID. He was doing well until August 2020 (at the age of 58 years), when he experienced tingling in the RUE associated with worsening cognitive difficulties. Repeat MRI showed 2 new lesions in the supratentorial regions. He was switched to oral cladribine without new clinical events or new radiological activity. *This case highlights that although rare, some patients have continued disease activity despite an older age and longer disease duration*.**(A)** Axial FLAIR sequences of brain MRI in April 2019 showing 2 new lesions (arrows) compared to prior MRI in 2017, and concomitant with a relapse (weakness and proprioceptive ataxia of the left upper extremity). **(B)** Axial FLAIR sequences of brain MRI in August 2020, 13 months after starting dimethyl fumarate, showing 2 new lesions (yellow arrows) compared to MRI in April 2019, with a decrease in size of the right posterior frontal lesion seen on prior MRI (green arrow) concomitant to a relapse: weakness of LUE and LLE.

#### Evidence of immunosenescence in MS

Telomere length shortening, a hallmark of biological aging of immune cells, is more pronounced in MS compared to age-matched controls (75). Shorter leukocyte telomere length is also associated with an increased risk of clinical progression over time (76). In line with this, the proportion of naïve T lymphocytes is reduced in MS compared to age-matched controls, and this alteration is observed even in pediatric MS cases (77). Antigen-experienced CD28^neg^ T cells exhibiting cytotoxic properties are observed in MS peripheral blood and CNS lesions (29). Other studies reported premature senescent expression patterns of age-sensitive immune markers by CD8 T lymphocytes from young MS patients (78). Pender et al. observed a reduction in the proportion of CD8 T cells producing interferon-gamma in response to autologous EBV infected cells in older patients compared with healthy donors (79). One group recently documented that the percentage of naïve CD4 T lymphocytes was lower, while the proportion of effector memory counterparts was higher in MS patients compared with healthy donors across ages (77). They also reported that the proportion of T lymphocytes expressing activation and cytotoxicity markers linked to aging is increased in MS patients (77). Finally, pro-inflammatory age-associated B cells are more frequent in MS patients before the age of 60 years than in age-matched controls (55).

#### Factors driving premature immunosenescence and neurodegeneration in MS

Numerous factors have been proposed as drivers of immunosenescence. Repeated antigen encounters, such as seen in autoimmune diseases and in chronic viral infections, accelerate immunosenescence (80, 81). Multiple MS risk or prognostic factors such as viral infections, smoking, obesity, and sedentary lifestyle can accelerate immunosenescence and CNS dysfunction.

Smoking and obesity are associated with increased markers of DNA damage and telomere shortening in peripheral blood cells (82). Obesity is associated with peripheral and CNS inflammation, lower synaptic plasticity, and accelerated brain atrophy (83). Obesity speeds up T cell immunosenescence, including thymic involution (84) and enhances the proportion of peripheral blood memory CD4 and CD8 T cells (85). In fact, the complications of obesity, e.g., the metabolic syndrome, are associated with a state of chronic inflammation (metaflammation) similar to inflammaging, suggesting overlapping mechanisms and causes between inflammaging and metaflammation (86).

Interestingly, exercise is one of the most effective anti-aging interventions; it has profound effects on the immune system and the CNS (87). Exercise increases thymic output, skew myeloid cells towards an anti-inflammatory phagocytosing phenotype, boosts immune responses to pathogens and limits clinical manifestations of latent viruses, autoimmunity and inflammation (36). In addition, exercise is associated with better brain microstructural integrity and lower retinal and hippocampal atrophy in patients with MS (88). Notably, exercise decreases CNS inflammation and promote remyelination in MS mouse models (89). In addition to exercise, effective anti-aging dietary/metabolic interventions, such as intermittent fasting (90), metformin (91), and methionine restriction (92), ameliorate inflammation, remyelination and disease course in MS and its animal models. Shared mechanisms between exercise and dietary interventions include a beneficial impact on gut microbiota composition. Gut dysbiosis is indeed observed upon aging and is considered to precede onset of multiple age-related comorbidities and contribute to immunosenescence (Figure 1C) (93). MS and other autoimmune diseases are associated with gut dysbiosis, which is considered to contribute to skew the immune system towards a pro-inflammatory response (94). The composition of the gut microbiota is further modulated by obesity, diet, exercise and DMTs (93). Interventions aimed at restoring a healthy gut microbiota environment are promising nonpharmacological avenues to improve age-related comorbidities, inflammation and subsequently MS outcomes.

## Confounders of clinical worsening with aging

The total burden of illnesses increases with age, leading to the higher prevalence of several common diseases such as hypertension, coronary heart disease, osteoarthritis, cancers, Alzheimer’s disease, among others. This increase also affects aging MS patients who suffer from an already lower than average health status. Comorbidities have an important functional impact independent of MS and may explain, at least in part, the heterogeneity in outcomes between individuals (11). The presence of comorbid disorders is particularly important in the interpretation of new symptoms in aging MS patients. Neurologists must ascertain whether a decline in function is attributable to worsening MS or comorbid illnesses, which has an impact on treatment strategies. Comorbidities also directly affect the MS course. In a 3-year longitudinal study, comorbidities significantly impacted clinical outcomes (specifically, patient-reported outcome measures and timed 25-foot walk scores) in a real-world MS cohort, and a cumulative effect with multiple comorbidities was observed (95). Cardiovascular comorbidities in particular may promote neurodegenerative processes, as observed in some studies showing accelerated brain atrophy among individuals with hypertension (96). In a retrospective US observational cohort, age-related comorbidities such as cardiovascular risk factors, osteoarthritis, osteoporosis, glaucoma, and cancer were highly prevalent in MS, particularly in patients older than 65 years (97). The presence of multiple comorbidities in an individual patient was also highly prevalent in this MS cohort (97).

**Figure 3:**
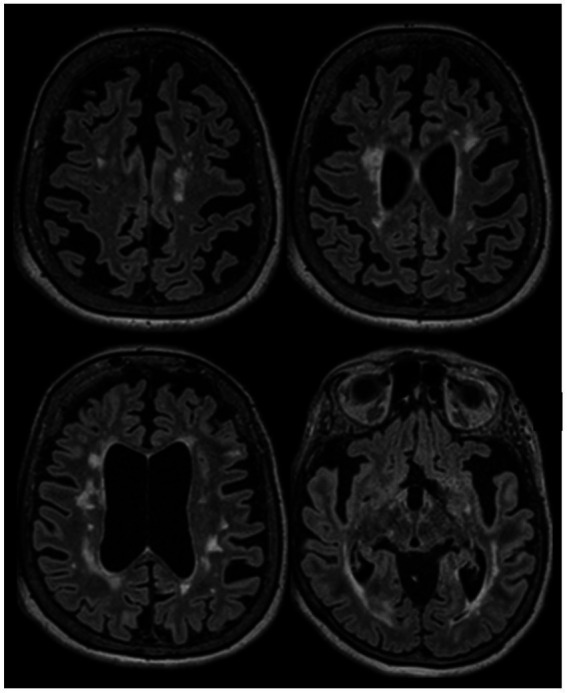
A 66-year-old woman was evaluated to establish care in our clinic. She was diagnosed with RRMS in 2002 after an episode compatible with sub-acute sensory myelitis. She was a participant in a Natalizumab trial, and was treated with this drug between 2003 and 2006. She experienced a relapse in 2007 after Natalizumab discontinuation, and was started on interferon beta1-a IM once a week between 2007 and 2015. She had a pseudo-relapse in 2014 in the context of acute illness. She did not report subacute symptoms since 2014, however she has noticed progressively worsening gait difficulties over the past few years, which she attributes to invalidating mechanical lower back pain related to severe spinal stenosis and degenerative disk disease. She continues to have flu-like symptoms after each interferon injection which last 12 to 24 hours. Her brain MRI in 2022 (at the age of 65) is stable compared to her MRIs between 2015 and 2019, except for a mild worsening of whole brain atrophy. After explaining the risks and benefits of treatment discontinuation, we decided to discontinue her interferon treatment. She has been clinically stable since. *This case highlights that treatment discontinuation is a reasonable option in older patients with long standing disease duration and no clinical/radiological activity*. Axial FLAIR sequences of brain MRI in July 2022 showing extensive lesions in the supratentorial regions which were stable in number and size compared to prior MRI in 2017, associated with diffuse brain atrophy.

### Cardiovascular comorbidities

Multiple sclerosis patients are at a higher risk than the general population to develop cardiovascular comorbidities such as hypertension, diabetes, dyslipidemia, ischemic heart disease, and cerebrovascular events (98). In a recent population-based retrospective matched cohort study from England, the risk of acute coronary syndrome, and cerebrovascular disease was approximately 30% higher in patients with MS than in the general population (99). A 3.5-fold increased hazard of all-cause mortality and a 1.5-fold increased hazard in cardiovascular disease-related mortality was also observed in this cohort (99). Similar results were observed in other cohorts (100). In a large population-based study using administrative data from four Canadian provinces, the incidence of diabetes in the MS population appears to be increasing more than in the general population (101). Interestingly, MS-related disability was associated with an increased risk of acute myocardial infarction in a Canadian MS cohort, which probably reflects sedentarity, lower exercise levels, and associated risk of obesity (100). The increased risk of cardiovascular events in MS might not be explained by a higher risk of cardiovascular risk factors alone, as MS cohorts were matched to controls after adjusting for age, sex, race, socioeconomic status, traditional risk factors, and antihypertensive treatments, statin use, and antiplatelets use (99, 100). It is postulated that chronic inflammation in MS and several other inflammatory diseases such as rheumatoid arthritis contribute to increasing the cardiovascular risk in addition to traditional risk factors, although treatment-related effects cannot be excluded (100, 102).

The radiological correlate of the effect of cardiovascular comorbidities on the CNS is reflected by the accumulation of sub-cortical white matter abnormalities often referred to as microvascular changes. The pathogenesis of lesion formation in this case results from endothelial injury, decreased perfusion in distal arterioles, ischemia, and disruption of the blood–brain barrier (103). Features of microvascular lesions on brain MRI include acute or sub-acute small subcortical infarcts seen on diffusion imaging, chronic lacunar infarcts mostly in the deep white and grey matter, sub-cortical white matter hyperintensities, enlarged perivascular spaces, microbleeds on susceptibility-weighted imaging, and global atrophy. Supratentorial MS lesions are typically ovoid periventricular, or juxtacortical. However, lesion formation in the sub-cortical regions is not uncommon with MS and the distinction can be difficult in practice (103). Differentiating microvascular lesions secondary to cerebral small vessel disease from new MS lesions is a key component in the evaluation of treatment response in older individuals with cardiovascular comorbidities. Although age-related whole brain and focal atrophy occur in all individuals with or without cardiovascular comorbidities, excess atrophy beyond what is expected from normal aging is seen in both MS and cerebrovascular disease. While MS-related atrophy is the main driver of global and focal brain volume loss in young individuals, the rate of normal aging increases and becomes the predominant contributor of atrophy after the age of 60 years (104). This has important implications when interpreting worsening atrophy in an older individual with long-standing MS.

### Osteoarticular comorbidities

Reduced bone mineral density is more frequent in patients with MS since corticosteroid use and reduced mobility are known risk factors for osteoporosis (105). Combined with the fact that the risk of falls is increased in long-standing MS, aging patients are predisposed to fractures. Osteoarthritis and lower back pain are common in aging MS patients, affecting around 20–25% of those older than 65 years (97). Osteoarticular-related mobility impairment and pain is important to distinguish from MS-related worsening and can be the main disabling symptom in some patients. However, aging individuals in general are also at higher risk of gait imbalance, falls, and osteo-articular disorders, even without known neurological diseases, hence the influence of aging, polypharmacy, and comorbidities on disability measures could be significant. As an important illustrative point, aging individuals without MS or other neurological conditions also demonstrate high level of disability on the Expanded Disability System Score (EDSS), the most commonly used measure of disability in MS. Lynch et al. report an EDSS ≥4 in a third of individuals without MS who are 55 years or older, associated with impairment in all functional system scores of the EDSS, except the cerebellar and visual components (106).

### Dementia

Both aging and long-standing MS independently impact cognitive functioning (107). Moreover, as patients age, several comorbid symptoms and disorders can contribute to patient-perceived cognitive impairment such as polypharmacy, poor sleep, depression, anxiety, and fatigue. With age, the risk of Alzheimer’s disease (AD) and related dementias, as well as vascular dementia, increases in the general population (and in individuals with MS), which can be difficult to distinguish from MS-related cognitive impairment (108). AD is common in individuals >65 years old, and its prevalence is increasing with longer life expectancy in the Western world. In a large US retrospective cohort using private claims data, Mahmoudi et al. found that the incidence of early-onset AD and related dementias diagnosis was higher in individuals with MS aged 45–64 years and >65 years compared to non-MS individuals after adjusting for key confounders (109). This data suggests that MS patients may be at a greater risk of AD and related dementias, but is largely confounded by the high probability of misdiagnosis in this setting (i.e:, MS-related condition vs. AD) (109). Indeed, the major obstacle in identifying coexisting AD in an aging MS patient is the challenge of diagnosing AD in general, since a definitive diagnosis relies on post-mortem histopathological confirmation. Rare case reports and case series of probable or definite AD in MS patients have been published (110). As an example, cortical lesions containing amyloid plaques and neurofibrillary tangles suggestive of AD were found in 11 out of 67 patients with long-standing inactive MS in an autopsy case series (15). In practice, the differential diagnosis relies on the identification of different cognitive phenotypes and, when available, the use of paraclinical testing such as PET imaging and CSF biomarkers. AD often presents with deficits in episodic and semantic memory, executive functioning, apraxia, and agnosia (cortical dementia) and evolves in most cases to moderate to severe dementia, whereas MS-related cognitive dysfunction often involves processing speed, verbal memory, and visual memory impairments and dementia is rarely seen (111). Comparing cognitive phenotypes between patients with MS, patients with amnestic mild cognitive impairment, and healthy individuals all aged 60–80 years, a study showed that MS patients had poorer performance on measures of processing speed, but better performance on cued memory, language, and executive function tests compared to patients with amnestic mild cognitive impairment (112). Vascular cognitive impairment secondary to microvascular changes is highly variable but is generally associated with poor executive function and impaired processing speed and can evolve slowly into an insidious sub-cortical dementia (108). Due to the high inter-individual variability of cognitive phenotypes particularly in MS and vascular-related cognitive impairments, the diagnosis remains a challenge in clinical practice.

### Frailty

Frailty is defined as a state of increased vulnerability resulting from aging-associated decline in reserve and function across multiple physiologic systems which occurs with age. Fried et al. operationally defined that frailty is reached with three out of five phenotypic criteria: low grip strength, low energy, slowed walking speed, low physical activity, and unintentional weight loss (113). A pre-frail stage, in which one or two criteria are present, identifies a subset at high risk of progressing to frailty. Frailty, which often accompanies aging in the general population, has a negative impact on the invalidity level in MS (114). Frailty carries an increased risk for poor health outcomes, including falls, incident disability, hospitalization, and mortality. To date, it is unclear if frailty is a reliable marker of handicap and morbidity related to MS.

## Treatment efficacy in aging MS patients

Pivotal randomized controlled trials leading to the approval and wide use of MS-specific DMTs have systematically excluded individuals >55 years old. However, as already mentioned, these individuals represent the majority of MS patients in real-world settings (1). The age discrepancy between individuals with MS included in regulatory trials and real-world clinical practice is concerning when considering treatment efficacy in this age group (115).

There is increasing evidence to support an age-dependent decrease in the efficacy of DMTs, which is consistent with the pathophysiological changes associated with aging combined with the fact that approved DMTs exert their efficacy via their anti-inflammatory properties. The majority of trials evaluating the efficacy of DMTs in progressive MS, particularly without evidence of clinical or radiological activity, have shown negative results. In a meta-analysis of all randomized clinical trials (including more than 28,000 participants) evaluating the efficacy of DMTs and using a complex statistical approach, Weideman et al. reported a strong decrease in the efficacy of DMTs on MS-related disability progression with advancing age, with chronological age explaining a large proportion of the variance in inhibition of disability progression (9). The regression model predicted no efficacy beyond the age of 53 years. Moreover, higher efficacy DMTs were superior to platform therapies only in patients younger than 40.5 years (9). However, in another large real-word study, high-efficacy DMTs appeared to be superior until the age of 54.2 years (116). Age was a better predictor of lower benefit on disability progression than baseline EDSS (9). Despite several limitations related to the representativeness of subjects included in trials, this meta-analysis highlights the differential efficacy of DMTs based on age. Conversely, another meta-analysis using data from 26 trials of 14 different DMTs showed no significant associations between age and efficacy in reduction of inflammatory activity markers (annualized relapse rate (ARR), new T2 lesions, and gadolinium-enhanced lesions) between active and comparator groups. This can be explained by the inclusion of patients with baseline disease activity in these trials, therefore not representing real-world patients with RRMS above a certain age. In a Canadian population-based observational study using linked administrative health data and including more than 19,000 MS patients, a protective effect of DMTs on hospitalization rate was observed in subjects <55 years but the risk was not significantly lowered in those >55 years (117).

Subgroup analyses of the comparative DMT effectiveness based on age were conducted in most phase 3 pivotal trials (115) and are summarized in Table 1. Most DMTs show little to no effect on disability progression in patients older than 40 years compared to comparator arms. A positive effect on markers of disease activity such as the ARR is seen in patients >40 years in several but not most trials (ex: Natalizumab trials AFFIRM and SENTINEL, Dimethyl fumarate trial DEFINE - but not in CONFIRM, and in the Peginterferon Beta-1a ADVANCE trial). Again, this can be explained by the inclusion of patients with baseline disease activity in most trials.

**Table 1:** Reported *post hoc* sub-group analysis from pivotal phase 3 trials of different DMTs based on age.

Treatment	Trial	Age-based effect on disease activity markers	Age-based effect on the risk of confirmed disability progression
Teriflunomide	TEMSO (118)	Significant reduction of the ARR in patients <38 and ≥38 years vs. placebo	Reduction of the risk of disability progression only in patients <38 years vs. placebo
Dimethyl fumarate	DEFINE (119) CONFIRM (120)	Significant reduction in the ARR in patients <40 and ≥ 40 years vs. placebo No significant reduction in the ARR in patients ≥40 years vs. glatiramer acetate	Reduction of the risk of disability progression only in patients <40 years vs. placebo No reduction in the risk of disability progression in both age groups (<40 and >40 years) vs. glatiramer acetate
Fingolimod	FREEDOMS (121)	No significant reduction in the ARR in patients ≥40 years vs. placebo	No reduction in the risk of disability progression in both age groups vs. placebo
Siponimod	EXPAND (122)	–	Significant reduction in the risk of disability progression in patients <50 and years ≥50 years vs. placebo
Ozanimod	SUNBEAM (123) and RADIANCE (124)	No significant reduction in the ARR in patients ≥40 years vs. IFN-b1a	No reduction in the risk of disability progression in both age groups vs. placebo vs. IFN-b1a
Cladribine	CLARITY (125)	Significant reduction in the odds of remaining free of disease activity in patients <40 and ≥40 years vs. placebo	Significant reduction in the risk of disability progression in patients <40 and years ≥40 years vs. placebo
Ocrelizumab	OPERA I and II (126) OROTARIO (127)	No significant reduction in the ARR in patients ≥40 years, but significant reduction in NEDA rates in both sub-groups vs. IFN-b1a Significant reduction in the ARR in patients <45 and ≥45 years vs. placebo	Significant reduction in the risk of disability progression in patients <40 and years ≥40 years vs. IFN-b1a Significant reduction in the risk of disability progression in patients <45 and ≥45 years vs. placebo with a notable trend to benefit younger subjects
Ofatumumab	ASCLEPIOS (128)	Significant reduction in the ARR in patients <40 and years ≥40 years vs. teriflunomide	Significant reduction in the risk of disability progression in patients <40 and years ≥40 years vs. teriflunomide
Natalizumab	AFFIRM and SENTINEL (129)	Significant reduction in the ARR in patients <40 and ≥40 years vs. placebo in AFFIRM and in combination with iINF-b1a vs. INF- b1a alone in SENTINEL	Reduction of the risk of disability progression only in patients <40 years vs. placebo in AFFIRM and in combination with INF-b1a vs. INF-b1a alone in SENTINEL

ARR, annualized relapse rate; INF, interferon; NEDA, no evidence of disease activity.

Siponimod and ocrelizumab were evaluated in slightly older populations. The phase 3 Siponimod clinical trial (EXPAND) included patients up to 60 years of age with SPMS (122). The mean age in this cohort was 48 years whereas, in other trials, the mean age varied between 33 and 38 years old (130). The majority (64%) of patients had no clinical relapse in the past 2 years and around half needed assistance for walking, therefore including an underrepresented population in previous trials. Siponimod was superior to placebo in reducing the risk of disability progression as measured by the EDSS (but not the timed 25-foot walk test), radiological activity, and percent brain volume loss compared to placebo. The effect on confirmed disability progression was seen in patients younger and older than 50 years (Poster presentation by Hua L. et al. at the American Academy of Neurology meeting in 2022, P5.002). CONSONNANCE, an ongoing open-label single-arm study evaluating the efficacy of ocrelizumab in patients with SPMS and PPMS, is the first trial including patients up to 65 years old (131). Primary outcome measures include No Evidence of Progression (NEP) defined as the absence of 24-weeks confirmed clinical progression (measured by a clinically significant increase in the EDSS score, the timed 25-foot walk test, or the nine-hole peg test) and No Evidence of Progression or Active Disease (NEPAD) defined as NEP plus the absence of relapses or radiological activity. The mean age in this study was 48.5 years. In the year-1 interim analysis, ocrelizumab was effective in maintaining NEP in >70% and NEPAD in approximately 58% of patients with SPMS and PPMS. The relatively high proportion of patients not meeting NEPAD at year one is explained by the therapeutic lag expected with the drug, as new/enlarging MRI lesions in the first 6 months were the main driver of NEPAD. However, this suggests that subjects included had some degree of inflammatory activity, therefore not representing real-world cohorts of patients with long-standing progressive MS.

## Treatment safety in aging MS patients

### Risks associated with disease-modifying therapies

As discussed in the first section of this paper, biological aging-related qualitative and quantitative changes in the immune system are associated with decreased ability to counter infections and cancers. Added to treatment-specific immunomodulation and immunosuppression, older individuals are at higher risk of adverse events with prolonged DMT use (13). As patients age, they might also be exposed to a higher number of DMTs with different mechanisms of action and this cumulative effect is not without risks. In general, older individuals are more prone to serious adverse events, in particular severe adverse events (132–135). An important example is the risk of progressive multifocal leukoencephalopathy (PML), mostly associated to the use of natalizumab but with other DMTs as well. Older age is an independent risk factor for developing PML. Older individuals are more likely to develop PML after a lower number of natalizumab infusions and have higher mortality rates (50, 136, 137). *De novo* infections and reactivation of latent viruses also occur more frequently in older individuals both in the general population and in MS. Particularly, the risk of varicella zoster virus associated to shingosine-1-phosphate receptor modulators, cladribine, and alemtuzumab increases with age (13). The risk of grade 3 lymphopenia with dimethyl fumarate use increases with age, and dimethyl fumarate-associated PML risk is related to severe lymphopenia (98). B-cell depleting therapies are associated with a higher risk of infections than interferon-based preparation, glatiramer acetate, fingolimod, and natalizumab, particularly in older individuals with comorbidities (138). The risk of serious infection with these therapies is partially correlated to the degree of associated hypogammaglobulinemia (139). This risk can be mitigated by monitoring immunoglobulin levels while on treatment. Reactivation of latent hepatitis B is a known risk of B-cell depleting therapies but does not seem to be affected by age. During the COVID-19 pandemic, MS patients on B-cell depleting therapies such as ocrelizumab and rituximab had worse outcomes than those on other DMTs, even after adjusting for age and other confounders (140, 141). Age, progressive MS phenotype, higher disability, and the presence of comorbidities were often associated with poorer Covid-19-related prognosis in MS cohorts (140–143). Importantly, vaccine responses are significantly blunted in patients on B-cell depleting therapies and fingolimod (144), added to an age-dependent decreased immune response even in healthy individuals (34).

Older individuals are also more susceptible to several non-infectious adverse events of DMTs. For example, hypertension is a potential adverse event of teriflunomide and fingolimod, and is also more frequent with aging. The negative chronotropic effects of fingolimod might also be age-dependent, at least in mice (145). Patients with type 2 diabetes, which becomes more frequent and more commonly associated with end-organ damage with aging, are more prone to fingolimod-related macular oedema. There are conflicting data regarding the risk of cancer in MS in general, with some studies (including a recent meta-analysis) reporting a lower risk of cancers in MS patients (146), and others a similar or slightly increased risk compared to the general population (147, 148). Cancer risk also increases with age and can be potentiated by the use of certain DMTs (148). For example, there is an increased risk of non-melanoma skin cancers with the use of fingolimod. B-cell depleting therapies, alemtuzumab, and natalizumab have also been associated with various cancers, but the evidence for causality is less robust. Despite the initial concern of increased carcinogenesis with cladribine, subsequent data showed no increase in risk of secondary malignancies (148). The overall cancer risk is probably not higher with exposure to IFN-b, glatiramer acetate, teriflunomide, and dimethyl fumarate (148–150).

### Risk of polypharmacy

Polypharmacy, commonly defined as the concomitant use of at least 5 medications, is observed in up to 35 to 50% of adults >65 years of age in North America (151, 152). Polypharmacy is a major public health concern globally, and is associated with an increased risk of drug–drug interactions and adverse events, lower quality of life, worsening disability and cognitive function, and increased hospitalization rates (152). Older adults with multiple comorbidities might be more prone to slowed drug metabolism, side effects, adverse events and drug interactions. Adverse events are estimated to be the 14th leading cause of morbidity and mortality in the world as per the WHO (153). Moreover, the estimated prevalence of hospitalizations due to drug interactions-related morbidity is around 1% (154). Patients with chronic diseases such as MS are at higher risk of polypharmacy, mainly secondarily to the use of symptomatic therapies. In a large Canadian population-based study, using administrative and pharmacy data in the universal healthcare setting of British Columbia, 28% of MS patients met the criteria of polypharmacy, and more than 2/3 of these were exposed to polypharmacy for more than 180 days in 2017 (155). Patients in the polypharmacy group had a significantly higher odd of hospitalizations compared to the non-polypharmacy group. Additionally, one in 20 MS patients were treated with ≥10 medications. Within those exposed to polypharmacy, 82% were older than 50 years. Compared to those aged <50 years, the odds of being exposed to polypharmacy was 2 times higher in MS patients aged 50–64 years, and more than three times higher in those ≥65 years. MS patients with 1–2 or ≥3 comorbidities had 3- and 6-times higher odds, respectively, of being exposed to polypharmacy. Interestingly, women and people with lower socio-economic status were also at higher risk of polypharmacy in this cohort. Anti-depressants followed by antiepileptics with analgesic properties (pregabalin, gabapentin, clonazepam), proton-pump inhibitors, lipid-lowering agents, centrally-acting muscle relaxants, ACE inhibitors, opioids, and thyroid medication, were the most commonly prescribed drugs in this study; this is consistent with a larger scale Medicare study in the US (156).

Polypharmacy is associated with poorer outcomes in MS patients. For instance, the risk of falls seems to be higher. In a *post hoc* analysis of data from two observational cohorts from the US and Australia, the adjusted odds of falling increased by 13% with each additional medication used (157). This increase was even more pronounced with centrally-acting drugs, specifically anti-depressants. In this study, the use of MS-specific DMTs decreased the risk of falls. In another prospective study, in which 85 MS patients were evaluated for depression, fatigue, self-reported cognitive functioning, and objective cognitive tests, those exposed to polypharmacy had increased fatigue and poorer self-reported cognitive functioning and performances on objective memory tests (158). People with MS are also more prone to potential drug–drug interactions which are more frequent in older individuals with a longer disease duration and higher EDSS scores (159).

The consequences of polypharmacy remain underrecognized among patients with MS and should be taken into account when evaluating older patients who are experiencing new or worsening symptoms. As suggested by Bourdette et al., polypharmacy should be highlighted in the problem list, when appropriate, to incite routine evaluation of medication lists (160). Specifically, the efficacy of MS symptomatic therapies should be periodically revised and discontinuation of treatment with no or little benefit should be encouraged while promoting non-pharmacological interventions to address pain, spasticity, poor sleep, and fatigue.

## Trends in DMT use in older MS patients

In practice, neurologists are often confronted with the decision to either maintain, escalate, de-escalate, or discontinue therapies in patients with long-standing disease who are 55 years or older. There are currently no evidence-based guidelines on treatment in this age group, and treatment decisions should remain individualized in a case-by-case approach. The European Academy of Neurology and American Academy of Neurology practice guidelines do not clearly address the indication of DMT de-escalation or discontinuation. Some patients with RRMS continue to have active disease despite their age and long disease duration, whereas a majority either have stable disease or evolve into a secondary progressive course as illustrated in the clinical vignettes (Figures 2,3).

### Treatment de-escalation

Little is known about de-escalation strategies in MS. The theoretical logic of de-escalation is based on the fact that the probability of disease activity is the highest in the first 5–10 years, and DMTs are mostly effective during this period (116). In a recent study, Vollmer et al. report a lower probability of disease activity in patients on higher-efficacy infusible DMTs vs. oral therapies until the age of 54.2 years, after which the difference in efficacy becomes non-significant (116). For this reason, de-escalation should be considered in older patients with stable disease, especially those at risk of serious adverse events (for ex: a patient with hypogammaglobulinemia on a B cell- depleting therapy). De-escalation can be done by switching to a lower efficacy DMT with more favorable safety profile before considering treatment discontinuation. However, whether the decrease in the risk of rebound disease activity is not counterbalanced by the safety and tolerability issues of cumulative exposure to DMTs is unclear. Another approach is de-escalation by interval extension for infusible DMTs (161, 162). The latter could offer the benefit of preserved efficacy with a reduced risk of adverse effects based on observations from the natalizumab and rituximab interval extension studies (161, 162). As proposed by Vollmer et al. de-escalation strategies developed to match the probability of disease activity across the lifespan need to be studied using randomized controlled trials (116).

### Treatment discontinuation

The risk of treatment discontinuation should take into account the annualized relapse rate and the presence or absence of radiological activity on MRI in the recent years. Continued progressive worsening despite treatment is not an uncommon reason to discontinue DMTs in patients with secondary progressive MS. DMT-specific considerations should also be factored in, for example, a careful evaluation of the risk of long-lasting immunosuppression with B-cell depleting therapies, alemtuzumab, and cladribine in infection-prone patients with advanced MS. Discontinuing DMT in older patients appears to have no effect on quality of life measures based on a three-center study comparing those outcomes between stoppers and stayers in a cohort of 600 MS patients above the age of 60 (163). Importantly, patients’ perspective on treatment discontinuation should be considered. Reluctance on treatment discontinuation is frequent in patients who have been stable for many years and are tolerating their therapies. A recent study evaluated patient-perspective on treatment discontinuation using a survey sent to patients from the North American Research Committee on Multiple Sclerosis (NARCOMS) registry who were ⩾45 years and on their most recent DMT for ⩾5 years (164). The mean age of respondents was approximately 56 years (164). In this study, 66.3% of respondents were unlikely or very unlikely to accept DMT discontinuation (164). In our experience, patients who are stable are usually more reluctant to treatment discontinuation compared to those with continued disability worsening.

Based on the observation of a lower benefit and potentially higher risk profile of DMT use in aging MS patients, recent research has focused on determining when and how to discontinue DMTs in older individuals. The DISCOMS (Discontinuation of Disease Modifying Therapies in MS) trial (NCT# 03073603) is the first randomized controlled trial evaluating the risk of disease activity after treatment discontinuation across 20 centers in the USA. The methodology of this trial was based on a non-inferiority analysis and the primary outcome was the combined measure of relapse and/or new T2 lesion on brain MRI. Other secondary and tertiary outcomes were also analyzed (6-months confirmed EDSS worsening, Symbol-Digit Modalities Test (SDMT) scores, and patient-reported outcome measures). Patients were randomized (1:1 by site) to either continue or discontinue their DMT. Clinical evaluation (relapse occurrence and EDSS scores), as well as MRI interpretation was performed by blinded raters. Mean follow-up time was 22.4 months. Included individuals (*n* = 259) were 55 years or older of any MS phenotype, had no relapse in the past 5 years, no new MRI lesions in the past 3 years, and were continuously treated with an approved DMT for at least 5 years, with the most recently used DMT for at least 2 years. The mean age of this cohort was 63 years, with a majority of women and RRMS phenotype. Most patients had longstanding (mean disease duration of 22.2 years) and stable (mean time since last documented relapse of 14 years) disease. Around 75% of subjects were treated with low-efficacy injectable therapies (interferon beta1a or 1b or glatiramer acetate) or teriflunomide. The combined occurrence of disease events was 4.69% vs. 12.21% (*p* = 0.521) meaning that non-inferiority was not demonstrated for this combined outcome. Relapses occurred in 0.78% of subjects in the group of patients who continued their DMT and 2.29% in the group who discontinued their DMT (*p* = 0.005), which implies non-inferiority of treatment discontinuation for this outcome. New T2 lesions occurred in 3.9% of subjects in the group of patients who continued their DMT and 10.69% in the group who discontinued their DMT (*p* = 0.422), which implies that non-inferiority was not demonstrated for this outcome. Importantly, these primary outcome events were not associated with worsened disability. Moreover, relapses were very rare in both groups, and most participants with relapses did not have a new lesion on MRI to corroborate the clinical event, suggesting the possibility of pseudo-relapses. New MRI lesions were rare and most had one new lesion only. EDSS progression was seen in about 11% of patients who continued their DMT and 12% of patients who discontinued their DMT, a difference that was not statistically significant. There was also no difference in SDMT scores and patient-reported outcome measures. The authors conclude that although DMT discontinuation is not inferior to continuing DMT in this population, non-inferiority of discontinuing DMT was not demonstrated (in other words: stopping treatment is not non-inferior to continuing treatment).An extension study of the DISCOMS trial is expected (NCT# 04754542; Corboy J et al. ECTRIMS 2022; EP1089). Two other randomized trials are also ongoing: the STOP-I-SEP trial (Disease Modifying Therapies Withdrawal in Inactive Secondary Progressive Multiple Sclerosis Patients Older Than 50 Years, NCT# 03653273, estimated study completion date in January 2028) and the DOT-MS trial (Discontinuing Disease-modifying Therapies in Stable Relapsing-Onset Multiple Sclerosis, NCT# 04260711, estimated study completion date in January 2024).

Other observational studies have evaluated safety of treatment discontinuation in patients with MS, specifically platform therapies. In a large multicenter study using data from the MS base registry in patients with at least 5 years of disease stability treated with INF-b1a/b or glatiramer acetate, and using propensity score matching, patients who pursued and those who stopped their DMT were compared (165). Patients in both groups had a similar relapse rate, but those who discontinued their DMT had a 50% higher hazard for disability progression than those who stayed on treatment, and this higher risk was mostly driven by patients who were stable prior to DMT discontinuation (165). In a small retrospective cohort (n = 69) predominantly treated with glatiramer acetate and interferon with stable disease for >2 years, patients who were < 45 years had a significantly shorter time to first clinical or radiological activity event compared to older patients (166). In a cohort of 221 patients with RRMS treated with either glatiramer acetate or interferon beta 1a/b who discontinued their treatment, Bsteh et al. retrospectively identified an age of 45 years or older at the time of DMT discontinuation, the absence of relapses for more than 4 years, and the absence of active lesion on MRI as independent predictors of absence of clinical activity after stopping DMT (167). Patients who were both older than 45 years and had no relapses in the past 4 years had a very low risk of relapses after stopping their DMT (167). Importantly, higher EDSS scores, an age older than 45 years, and longer disease duration at treatment discontinuation were all associated with a higher risk of disability progression in this study after discontinuation (167). The same group evaluated the performance of a composite score taking into account age, radiological activity and the duration of disease stability prior to DMT discontinuation, and showed that patients with a high composite score had an 85% probability of recurrence of disease activity in the next 5 years after stopping their treatment (168). Similar results were observed in another observational study using propensity score matching to compare patients who stayed or stopped their injectable DMT (169). The mean age of this cohort was 54 years and all included patients were at least 50 years and did not have a relapse in the past 3 years (169). Stoppers did not have a higher likelihood of relapse or EDSS progression, but had a higher risk of reaching an EDSS of 6.0 (169). In another recent multi-center retrospective observational study from Jakimovsky et al. DMT discontinuation was associated with non-relapse disability progression, or PIRA, independently of prior stable disease and age, specifically in patients with an EDSS >6.0 (170). In this study, DMT discontinuation triggered *de novo* disability worsening in previously stable patients with both RRMS and SPMS (170). Taken altogether, the results of these studies corroborate the hypothesis that PIRA is the main driver of disability along the disease course and that DMTs do not halt or alter this neurodegenerative process once it is ongoing, but may play a role in controlling subclinical inflammation even at a later age, specifically in patients with no clear evidence of progressive disability worsening while on treatment and those in the so-called “transition phase.”

Data on higher-efficacy DMT discontinuation after a certain age is scarce but yet less reassuring. A two-center study in France from Chappuis et al. retrospectively evaluated the risks of disease activity after platform (glatiramer acetate, interferon beta-1a/b, teriflunomide) and higher-efficacy (fingolimod, rituximab, natalizumab) DMT discontinuation in 232 patients who were older than 45 years (171). Median age in this cohort was around 53 years, median disease duration was 15.8 years, and mean EDSS was 3.8 at DMT discontinuation (171). Most patients did not have a relapse in the past year, but around 25% of those on higher-efficacy DMT and 16% of those on platform DMT did have clinical or radiological activity in the past 3 years (171). Nearly 40% of patients were classified as having SPMS. Importantly, 61.2% of patients who stopped their higher-efficacy DMT had progressive MS (171). A 6% relapse risk in the year after discontinuation was observed for the group on a platform DMTs, 9% for those on fingolimod and 43% for those on natalizumab was observed, peaking in the first 3 months after stopping fingolimod or natalizumab, while no patient had disease activity after stopping Rituximab (171). Hence, the well-established rebound effect after natalizumab discontinuation does not only occur in younger individuals with active relapsing–remitting disease, and remains a significant risk when considering stopping this treatment in older patients, even those with a secondary-progressive disease course. Results from a smaller prospective observational study are also in line with this observation: in 15 patients with a mean age of 50 years and stable disease for the past 5 years who discontinued natalizumab, all experienced disease activity after a mean follow-up period of 19 months (172). Specifically, 10 patients had a recrudescence of clinical or radiological activity whereas 5 had rebound activity (4 out 5 where >50 years old). In conclusion, discontinuing natalizumab specifically is associated with a significant risk of disease activity at any age and should prompt early switching to another DMT. In regards to fingolimod, the risk of disease activity or rebound activity after discontinuation appears to be lower and more age-dependent compared to Natalizumab, yet present. For instance, a single-center retrospective study looking at all patients who discontinued fingolimod for more than 6 months found than an age > 50 years did not significantly decrease the risk of recurrence of disease activity even though it occurred less frequently in this age group compared to their younger counterparts. Specifically, 11/128 patients were older than 60 years and none of them had recurrence of disease activity after stopping fingolimod (173). In the previously mentioned French study, 2 patients who were older than 60 years experienced recurrence of disease activity after stopping fingolimod (171). Discontinuing fingolimod should therefore also be considered cautiously at any age. There is little evidence on the risk of disease activity reactivation or rebound activity after discontinuation of B-cell depleting therapies. Nevertheless, this category of DMT seems to exert a prolonged effect without a significant risk of rebound of clinical or radiological activity. In a large single-center Swedish cohort treated with rituximab, 808 patients were retrospectively identified, only 92 (11%) had discontinued treatment mostly due pregnancy, adverse events, stable disease, and other reasons (174). There was no difference in age, disease duration, number of previous DMT, and EDSS at rituximab start between those who stayed on and those who stopped rituximab (174). After rituximab discontinuation, 3/92 patients had a relapse and 4/92 had new T2 lesions (one of which had both) at least 3 months after treatment stop (174). Although some patients who discontinued rituximab were started on another DMT, disease activity was rare even in those who stayed off therapy (174). In the French cohort, 9 patients discontinued Rituximab after the age of 45 and none had a relapse after a mean follow-up time of 1.6 years (171). Based on these observations, disease activity appears to remain suppressed long after B-cell depleting therapies discontinuation. Whether this DMT category can be used as an induction therapy and later discontinued safely without an alternative DMT is not clear but would be an interesting treatment approach when considering long-term treatment planning.

In conclusion, there is growing evidence to suggest that treatment discontinuation is relatively safe after the age of 55–60 years in individuals with long-standing and stable disease on platform therapies. Recurrence of disease activity however remains a risk, specifically for patients on fingolimod and natalizumab, and relapse recovery declines with age (175). Clinicians need tools to stratify the risk of disease reactivation to guide clinical decisions, such as the Vienna Innsbruck DMT discontinuation score based on age, activity on MRI, and duration in stable course (VIAADISC score) developed by Bsteh et al. for patients on platform DMTs (168). Predictive scores should be developed for other DMTs, specially for higher-efficacy DMTs as well as for teriflunomide and dimethyl fumarate for which data on discontinuation risk is scarce. The difficulty in developing such tools resides in the high interindividual variability of MS phenotypes and in the lack of predictability and intraindividual variability of disease activity across the disease course. Moreover, we need more time to assess long-term effects of newer DMTs. There are inherent differences in the risk of disease reactivation and the risk of rebound activity with some DMTs after discontinuation and conversely a potential for induction properties of others such as B-cell depleting therapies and cladribine. Finally, it is essential to consider the patients’ perspective when making such decisions; patient-reported outcome measures can guide clinicians understand how treatment decisions affect patients’ quality of life (163). For now, the decision to discontinue or de-escalate DMTs should be taken in the context of each individual with a clear explanation of the risk–benefit balance to the patient, taking into account treatment-related morbidity and direct and indirect treatment-related costs.

## Conclusion and recommendations

Caring for patients with long-standing MS is a quotidian and complex task for their health care providers. Several concerns must be integrated in treatment choices, including the disease process itself and its multiple sequelae, comorbidities, and measure of general health such as frailty. Development of clinical deterioration should be viewed as a potential complication from these several factors, and the management plan should be tailored accordingly. As patients age and transition to a predominantly progressive form, their needs increase as they accumulate symptoms such as weakness, ataxia, spasticity, cognitive impairment, pain, sphincteric and sexual dysfunctions, visual symptoms, sleep problems, and fatigue. Hence, management can become complex and requires frequent adjustments. To optimize health outcomes in this population, multidisciplinary care should be the cornerstone of management (176). Physical and occupational therapists, social workers, psychologists, speech therapists with an expertise in cognition, urologists, and physiatrists are all essential players in the treatment of long-standing MS, and can help patients to maintain their autonomy and quality of life (176). Involving patients in their own care can increase empowerment and coping abilities. In this regard, promoting physical activity, healthier life habits, weight control and good nutrition can delay disease progression and result in a higher sense of wellness. Unfortunately, MS care units remain a luxury in many regions, even in developed countries. As an example, using a survey targeting health care providers across Canada to assess models of MS care, Marrie et al. found that nearly half of MS clinics report an insufficient number of specialized neurologists, and nearly 70% report an insufficient number of non-physician providers (177). Sadly, a majority of clinics had wait times longer than 3 months for patients to be seen by the different providers of the multidisciplinary team (177). Another important aspect of MS management is recognizing polypharmacy and deprescribing when possible, as aging patients with MS often end up with several symptomatic therapies with additive side effects. Polypharmacy is an under-recognized problem and has an additive effect on MS symptoms particularly fatigue, cognitive impairment, and fall risk (160). As discussed throughout this review, the natural evolution of the disease, the shift of pathophysiological processes, the probable decreased efficacy of DMTs after the age of 55 years (supported by real-word data, clinical observations, and the DISCOMS trial), and the safety concerns in this age group, support the rationale of considering DMT de-escalation and discontinuation in older patients with stable disease, particularly those on platform DMTs (178). Until we have more reassuring data, careful monitoring for recurrence of disease activity after discontinuation is prudent (178). However, the evidence to support this practice is still scarce and there are currently no guidelines on treatment discontinuation, although several consensus groups have published recommendations along these lines (179, 180).

In this regard, recommendations regarding treatment approaches in individuals after the age of 55 years may include the following, until more evidence-based data become available and practice guidelines are developed:

- The benefits and risks of DMTs should be reassessed and discussed with patients periodically taking into account their age, disease duration, clinical and radiological activity in the past few years, rate of disability accrual, comorbidities, and patient preferences. Treatment decisions should hence be individualized in a case-by-case approach.
- DMT discontinuation could be considered in individuals with long standing and stable disease on platform DMTs who are older than 55 years, especially in those older than 60 years.
- The benefit of platform therapies such as interferon beta-1a or b and teriflunomide in individuals with long standing disease after the age of 55 is questionable, and since there is no reported risk of rebound disease activity after discontinuation of these therapies, they could be safely discontinued with careful monitoring in most cases.
- In general, de-escalation could be considered after the age of 55 in patients who have been on high-efficacy DMTs for many years. Although the benefit of fingolimod and natalizumab in this population is questionable, the risk of breakthrough disease activity or rebound activity is non-negligible. De-escalation can be used as a strategy to mitigate this risk.

o Switching to lower efficacy DMTs such as teriflunomide, interferon-based preparation, or glatiramer acetate before considering treatment discontinuation could be an option.
o Another approach could be de-escalation by interval extension for therapies such as natalizumab and B cell-depleting therapies, although whether the risk of rebound activity is sufficiently mitigated with this approach is unclear.
o In patients with recent disease activity for whom natalizumab or fingolimod must be discontinued (e.g., lymphopenia, positive JCV serology), switching to other high-efficacy “induction” therapies such as cladribine or B-cell therapies can be useful in selected cases. These DMTs have more prolonged immunosuppressive effects and do not seem to be associated with rebound effects when stopped, although an additive effect on the risk of PML should be explained to patients.

- At each visit, symptomatic therapies should be reviewed and ineffective medications discontinued. Instead, non-pharmacological interventions, such as aerobic exercise and good sleep hygiene to improve fatigue or stretching to counter spasticity must be encouraged.
- General measures of wellness should be optimized by promoting physical activity and adequate nutrition, optimization of comorbidity management, and promotion of age-specific preventive measures
- Developing outcome measures that are adapted to aging individuals to detect MS-related handicap and appropriately identifying confounders is key to evaluating treatment response and optimally address drivers of disability progression, whether related to MS or not. The EDSS, the most commonly used scale of disability in MS, might not be the ideal tool in this population, as higher scores are associated with older age and polypharmacy, even when used on older individuals who do not have MS (106).

## Author contributions

GM: conception and design of manuscript, data review and interpretation, drafting the manuscript, and reviewing the manuscript for intellectual content. CL and NA: drafting the manuscript and reviewing the manuscript for intellectual content. MG, JG, and AP: reviewing the manuscript for intellectual content. PD: conception and design of manuscript, data review and interpretation, and reviewing the manuscript for intellectual content. All authors contributed to the article and approved the submitted version.

## Conflict of interest

GM has served on advisory boards for Genentech-Roche, Novartis, Mercks, and Biologix, received speaker fees from Biologix, Mercks, and Novartis, and participated in educational activities for Neurology Live and John Hopkin’s e-Litterature Review. CL has served on scientific advisory boards and/or as speaker for EMD-Serono, Biogen, Bristol-Myers Squibb, Roche, Novartis, Actelion, FindTx and Sanofi-Genzyme and has received a Grant for Multiple Sclerosis Innovation from Merck/EMD-Serono.

The remaining authors declare that the research was conducted in the absence of any commercial or financial relationships that could be construed as a potential conflict of interest.

## Publisher’s note

All claims expressed in this article are solely those of the authors and do not necessarily represent those of their affiliated organizations, or those of the publisher, the editors and the reviewers. Any product that may be evaluated in this article, or claim that may be made by its manufacturer, is not guaranteed or endorsed by the publisher.

## References

[1] Wallin MT Culpepper WJ Campbell JD Nelson LM Langer-Gould A Marrie RA. The prevalence of MS in the United States: a population-based estimate using health claims data. Neurology. (2019) 92:e1029–40. doi: 10.1212/WNL.0000000000007035, PMID: 30770430 PMC6442006

[2] Marrie RA Yu N Blanchard J Leung S Elliott L. The rising prevalence and changing age distribution of multiple sclerosis in Manitoba. Neurology. (2010) 74:465–71. doi: 10.1212/WNL.0b013e3181cf6ec0, PMID: 20071664

[3] Kappos L Wolinsky JS Giovannoni G Arnold DL Wang Q Bernasconi C. Contribution of relapse-independent progression vs relapse-associated worsening to overall confirmed disability accumulation in typical relapsing multiple sclerosis in a pooled analysis of 2 randomized clinical trials. JAMA Neurol. (2020) 77:1132–40. doi: 10.1001/jamaneurol.2020.1568, PMID: 32511687 PMC7281382

[4] Lublin FD Häring DA Ganjgahi H Ocampo A Hatami F Čuklina J. How patients with multiple sclerosis acquire disability. Brain. (2022) 145:3147–61. doi: 10.1093/brain/awac016, PMID: 35104840 PMC9536294

[5] Kuhlmann T Moccia M Coetzee T Cohen JA Correale J Graves J. Multiple sclerosis progression: time for a new mechanism-driven framework. Lancet Neurol. (2023) 22:78–88. doi: 10.1016/S1474-4422(22)00289-7, PMID: 36410373 PMC10463558

[6] Zeydan B Kantarci OH. Impact of age on multiple sclerosis disease activity and progression. Curr Neurol Neurosci Rep. (2020) 20:1–7. doi: 10.1007/s11910-020-01046-2 32458308

[7] Zeydan B Kantarci OH. Progressive forms of multiple sclerosis. Neurol Clin. (2018) 36:163–71. doi: 10.1016/j.ncl.2017.08.006, PMID: 29157397

[8] Tutuncu M Tang J Zeid NA Kale N Crusan DJ Atkinson EJ. Onset of progressive phase is an age-dependent clinical milestone in multiple sclerosis. Mult Scler J. (2013) 19:188–98. doi: 10.1177/1352458512451510, PMID: 22736750 PMC4029334

[9] Weideman AM Tapia-Maltos MA Johnson K Greenwood M Bielekova B. Meta-analysis of the age-dependent efficacy of multiple sclerosis treatments. Front Neurol. (2017) 8:1–12. doi: 10.3389/fneur.2017.00577 29176956 PMC5686062

[10] Yu N Tennakoon A Cossoy M Marriott J Elliott L Marrie RA. Comorbidity increases the risk of hospitalizations in multiple sclerosis. Neurology. (2014) 84:350–8. doi: 10.1212/wnl.0000000000001187 25540309 PMC4336005

[11] Marrie RA. Comorbidity in multiple sclerosis: implications for patient care. Nat Rev Neurol. (2017) 13:375–82. doi: 10.1038/nrneurol.2017.33, PMID: 28303911

[12] Macaron G Cohen JA. Integrating multiple sclerosis guidelines into practice. Lancet Neurol. (2018) 17:658–60. doi: 10.1016/S1474-4422(18)30248-5, PMID: 30033052

[13] Schweitzer F Laurent S Fink GR Barnett MH Reddel S Hartung HP. Age and the risks of high-efficacy disease modifying drugs in multiple sclerosis. Curr Opin Neurol. (2019) 32:305–12. doi: 10.1097/WCO.0000000000000701, PMID: 30985373

[14] Hametner S Wimmer I Haider L Pfeifenbring S Bruck W Lassmann H. Iron and neurodegeneration in the multiple sclerosis brain. Ann Neurol. (2013) 74:848–61. doi: 10.1002/ana.23974, PMID: 23868451 PMC4223935

[15] Frischer JM Bramow S Dal-Bianco A Lucchinetti CF Rauschka H Schmidbauer M. The relation between inflammation and neurodegeneration in multiple sclerosis brains. Brain. (2009) 132:1175–89. doi: 10.1093/brain/awp070, PMID: 19339255 PMC2677799

[16] Howell OW Reeves CA Nicholas R Carassiti D Radotra B Gentleman SM. Meningeal inflammation is widespread and linked to cortical pathology in multiple sclerosis. Brain. (2011) 134:2755–71. doi: 10.1093/brain/awr182, PMID: 21840891

[17] Mahad DH Trapp BD Lassmann H. Pathological mechanisms in progressive multiple sclerosis. Lancet Neurol. (2015) 14:183–93. doi: 10.1016/S1474-4422(14)70256-X, PMID: 25772897

[18] Lassmann H. The contribution of neuropathology to multiple sclerosis research. Eur J Neurol. (2022) 29:2869–77. doi: 10.1111/ene.15360, PMID: 35427431 PMC9544263

[19] Goldschmidt T Antel J König FB Brück W Kuhlmann T. Remyelination capacity of the MS brain decreases with disease chronicity. Neurology. (2009) 72:1914–21. doi: 10.1212/WNL.0b013e3181a8260a, PMID: 19487649

[20] Chang A Staugaitis SM Dutta R Batt CE Easley KE Chomyk AM. Cortical remyelination: a new target for repair therapies in multiple sclerosis. Ann Neurol. (2012) 72:918–26. doi: 10.1002/ana.23693, PMID: 23076662 PMC3535551

[21] Bodini B Veronese M García-Lorenzo D Battaglini M Poirion E Chardain A. Dynamic imaging of individual remyelination profiles in multiple sclerosis. Ann Neurol. (2016) 79:726–38. doi: 10.1002/ana.24620, PMID: 26891452 PMC5006855

[22] Kolb H Absinta M Beck ES Ha SK Song Y Norato G. 7T MRI differentiates remyelinated from demyelinated multiple sclerosis lesions. Ann Neurol. (2021) 90:612–26. doi: 10.1002/ana.26194, PMID: 34390015 PMC9291186

[23] Waubant E Lucas R Mowry E Graves J Olsson T Alfredsson L. Environmental and genetic risk factors for MS: an integrated review. Ann Clin Transl Neurol. (2019) 6:1905–22. doi: 10.1002/acn3.50862, PMID: 31392849 PMC6764632

[24] Shams H Shao X Santaniello A Kirkish G Harroud A Ma Q. Polygenic risk score association with multiple sclerosis susceptibility and phenotype in Europeans. Brain. (2023) 146:645–56. doi: 10.1093/brain/awac092, PMID: 35253861 PMC10169285

[25] Golden LC Itoh Y Itoh N Iyengar S Coit P Salama Y. Parent-of-origin differences in DNA methylation of X chromosome genes in T lymphocytes. Proc Natl Acad Sci U S A. (2019) 116:26779–87. doi: 10.1073/pnas.1910072116, PMID: 31822606 PMC6936674

[26] Voskuhl RR. The effect of sex on multiple sclerosis risk and disease progression. Mult Scler. (2020) 26:554–60. doi: 10.1177/1352458519892491, PMID: 31965884 PMC7160019

[27] Giefing-Kröll C Berger P Lepperdinger G Grubeck-Loebenstein B. How sex and age affect immune responses, susceptibility to infections, and response to vaccination. Aging Cell. (2015) 14:309–21. doi: 10.1111/acel.12326, PMID: 25720438 PMC4406660

[28] Baranzini S Sawcer S. International multiple sclerosis genetics consortium. Genetic analysis of multiple sclerosis severity identifies a novel locus and implicates CNS resilience as a major determinant of outcome. Res Sq. (2022). doi: 10.21203/rs3.rs-1723574/v1

[29] Larochelle C Uphaus T Prat A Zipp F. Secondary progression in multiple sclerosis: neuronal exhaustion or distinct pathology? Trends Neurosci. (2016) 39:325–39. doi: 10.1016/j.tins.2016.02.001, PMID: 26987259

[30] López-Otín C Blasco MA Partridge L Serrano M Kroemer G. Hallmarks of aging: an expanding universe. Cells. (2023) 186:243–78. doi: 10.1016/j.cell.2022.11.001, PMID: 36599349

[31] Rébillard R-M Charabati M Grasmuck C Filali-Mouhim A Tastet O Brassard N. Identification of SARS-CoV-2-specific immune alterations in acutely ill patients. J Clin Invest. (2021) 131:e145853. doi: 10.1172/JCI145853, PMID: 33635833 PMC8262478

[32] Aragon L Iribarren-López A Alberro A Iparraguirre L von Wichmann M Marimon JM. Immune cell population and cytokine profiling suggest age dependent differences in the response to SARS-CoV-2 infection. Front Aging. (2023) 4:1108149. doi: 10.3389/fragi.2023.1108149, PMID: 36861136 PMC9968858

[33] Froidure A Mahieu M Hoton D Laterre PF Yombi JC Koenig S. Short telomeres increase the risk of severe COVID-19. Aging (Albany NY). (2020) 12:19911–22. doi: 10.18632/aging.104097, PMID: 33104521 PMC7655194

[34] Müller L Andrée M Moskorz W Drexler I Walotka L Grothmann R. Age-dependent immune response to the Biontech/Pfizer BNT162b2 coronavirus disease 2019 vaccination. Clin Infect Dis. (2021) 73:2065–72. doi: 10.1093/cid/ciab381, PMID: 33906236 PMC8135422

[35] Dema M Eixarch H Villar LM Montalban X Espejo C. Immunosenescence in multiple sclerosis: the identification of new therapeutic targets. Autoimmun Rev. (2021) 20:102893. doi: 10.1016/j.autrev.2021.102893, PMID: 34237417

[36] Duggal NA Niemiro G Harridge SDR Simpson RJ Lord JM. Can physical activity ameliorate immunosenescence and thereby reduce age-related multi-morbidity? Nat Rev Immunol. (2019) 19:563–72. doi: 10.1038/s41577-019-0177-9, PMID: 31175337

[37] Atkinson JR Jerome AD Sas AR Munie A Wang C Ma A. Biological aging of CNS-resident cells alters the clinical course and immunopathology of autoimmune demyelinating disease. JCI Insight. (2022) 7:e158153. doi: 10.1172/jci.insight.158153, PMID: 35511417 PMC9309055

[38] Godeanu S Clarke D Deftu A-F Popa-Wagner A Bălșeanu AT. Microglial morphology in the somatosensory cortex across lifespan. A quantitative study. Dev Dyn Off Publ Am Assoc Anat. (2023). doi: 10.1002/dvdy.582, PMID: [Online ahead of print]. 36883224

[39] Streit WJ Sammons NW Kuhns AJ Sparks DL. Dystrophic microglia in the aging human brain. Glia. (2004) 45:208–12. doi: 10.1002/glia.10319, PMID: 14730714

[40] de Haan G Lazare SS. Aging of hematopoietic stem cells. Blood. (2018) 131:479–87. doi: 10.1182/blood-2017-06-746412, PMID: 29141947

[41] Hartsock RJ Smith EB Petty CS. Normal variations with aging of the amount of hematopoietic tissue in bone marrow from the anterior iliac crest. A study made from 177 cases of sudden death examined by necropsy. Am J Clin Pathol. (1965) 43:326–31. doi: 10.1093/ajcp/43.4.326, PMID: 14275849

[42] Thomas R Wang W Su D-M. Contributions of age-related thymic involution to immunosenescence and inflammaging. Immun Ageing. (2020) 17:2. doi: 10.1186/s12979-020-0173-8 31988649 PMC6971920

[43] de Mol J Kuiper J Tsiantoulas D Foks AC. The dynamics of B cell aging in health and disease. Front Immunol. (2021) 12:733566. doi: 10.3389/fimmu.2021.733566, PMID: 34675924 PMC8524000

[44] De Maeyer RPH Chambers ES. The impact of ageing on monocytes and macrophages. Immunol Lett. (2021) 230:1–10. doi: 10.1016/j.imlet.2020.12.003, PMID: 33309673

[45] Gounder SS Abdullah BJJ Radzuanb NEIBM Zain FDBM Sait NBM Chua C. Effect of aging on NK cell population and their proliferation at ex vivo culture condition. Anal Cell Pathol (Amst). (2018) 2018:1–6. doi: 10.1155/2018/7871814, PMID: 30175033 PMC6098903

[46] Thomas AL Lehn MA Janssen EM Hildeman DA Chougnet CA. Naturally-aged microglia exhibit phagocytic dysfunction accompanied by gene expression changes reflective of underlying neurologic disease. Sci Rep. (2022) 12:19471. doi: 10.1038/s41598-022-21920-y 36376530 PMC9663419

[47] Sierra A Gottfried-Blackmore AC McEwen BS Bulloch K. Microglia derived from aging mice exhibit an altered inflammatory profile. Glia. (2007) 55:412–24. doi: 10.1002/glia.20468, PMID: 17203473

[48] Bieri G Schroer AB Villeda SA. Blood-to-brain communication in aging and rejuvenation. Nat Neurosci. (2023) 26:379–93. doi: 10.1038/s41593-022-01238-8, PMID: 36646876

[49] Sun X Nguyen T Achour A Ko A Cifello J Ling C. Longitudinal analysis reveals age-related changes in the T cell receptor repertoire of human T cell subsets. J Clin Invest. (2022) 132:e158122. doi: 10.1172/JCI158122, PMID: 35708913 PMC9433102

[50] Mills EA Mao-draayer Y. Aging and lymphocyte changes by immunomodulatory therapies impact PML risk in multiple sclerosis patients. Mult Scler J. (2018) 24:1014–22. doi: 10.1177/1352458518775550, PMID: 29774781 PMC6013383

[51] Han S Georgiev P Ringel AE Sharpe AH Haigis MC. Age-associated remodeling of T cell immunity and metabolism. Cell Metab. (2023) 35:36–55. doi: 10.1016/j.cmet.2022.11.005, PMID: 36473467 PMC10799654

[52] Bharath LP Agrawal M McCambridge G Nicholas DA Hasturk H Liu J. Metformin enhances autophagy and normalizes mitochondrial function to alleviate aging-associated inflammation. Cell Metab. (2020) 32:44–55.e6. doi: 10.1016/j.cmet.2020.04.015, PMID: 32402267 PMC7217133

[53] Fukushima Y Minato N Hattori M. The impact of senescence-associated T cells on immunosenescence and age-related disorders. Inflamm Regen. (2018) 38:24. doi: 10.1186/s41232-018-0082-9 30603051 PMC6304761

[54] Mouat IC Goldberg E Horwitz MS. Age-associated B cells in autoimmune diseases. Cell Mol Life Sci. (2022) 79:402. doi: 10.1007/s00018-022-04433-9 35798993 PMC9263041

[55] Claes N Fraussen J Vanheusden M Hellings N Stinissen P van Wijmeersch B. Age-associated B cells with proinflammatory characteristics are expanded in a proportion of multiple sclerosis patients. J Immunol. (2016) 197:4576–83. doi: 10.4049/jimmunol.1502448, PMID: 27837111

[56] Fessler J Angiari S. The role of T cell senescence in neurological diseases and its regulation by cellular metabolism. Front Immunol. (2021) 12:706434. doi: 10.3389/fimmu.2021.706434, PMID: 34335619 PMC8317490

[57] Wang Q Zhan Y Pedersen NL Fang F Hägg S. Telomere length and all-cause mortality: a meta-analysis. Ageing Res Rev. (2018) 48:11–20. doi: 10.1016/j.arr.2018.09.002, PMID: 30254001

[58] Yousefzadeh MJ Flores RR Zhu Y Schmiechen ZC Brooks RW Trussoni CE. An aged immune system drives senescence and ageing of solid organs. Nature. (2021) 594:100–5. doi: 10.1038/s41586-021-03547-7, PMID: 33981041 PMC8684299

[59] Villeda SA Plambeck KE Middeldorp J Castellano JM Mosher KI Luo J. Young blood reverses age-related impairments in cognitive function and synaptic plasticity in mice. Nat Med. (2014) 20:659–63. doi: 10.1038/nm.3569, PMID: 24793238 PMC4224436

[60] Villeda SA Luo J Mosher KI Zou B Britschgi M Bieri G. The ageing systemic milieu negatively regulates neurogenesis and cognitive function. Nature. (2011) 477:90–4. doi: 10.1038/nature10357, PMID: 21886162 PMC3170097

[61] Mayne K White JA McMurran CE Rivera FJ de la Fuente AG. Aging and neurodegenerative disease: is the adaptive immune system a friend or foe? Front Aging Neurosci. (2020) 12:572090. doi: 10.3389/fnagi.2020.572090, PMID: 33173502 PMC7538701

[62] Thome AD Faridar A Beers DR Thonhoff JR Zhao W Wen S. Functional alterations of myeloid cells during the course of Alzheimer’s disease. Mol Neurodegener. (2018) 13:61. doi: 10.1186/s13024-018-0293-1, PMID: 30424785 PMC6233576

[63] Minhas PS Latif-Hernandez A McReynolds MR Durairaj AS Wang Q Rubin A. Restoring metabolism of myeloid cells reverses cognitive decline in ageing. Nature. (2021) 590:122–8. doi: 10.1038/s41586-020-03160-0, PMID: 33473210 PMC8274816

[64] das MM Godoy M Chen S Moser VA Avalos P Roxas KM. Young bone marrow transplantation preserves learning and memory in old mice. Commun Biol. (2019) 2:73. doi: 10.1038/s42003-019-0298-5, PMID: 30820468 PMC6382867

[65] Ruckh JM Zhao J-W Shadrach JL van Wijngaarden P Rao TN Wagers AJ. Rejuvenation of regeneration in the aging central nervous system. Cell Stem Cell. (2012) 10:96–103. doi: 10.1016/j.stem.2011.11.019, PMID: 22226359 PMC3714794

[66] Jin W-N Shi K He W Sun JH van Kaer L Shi FD. Neuroblast senescence in the aged brain augments natural killer cell cytotoxicity leading to impaired neurogenesis and cognition. Nat Neurosci. (2021) 24:61–73. doi: 10.1038/s41593-020-00745-w, PMID: 33257875

[67] Dulken BW Buckley MT Navarro Negredo P Saligrama N Cayrol R Leeman DS. Single-cell analysis reveals T cell infiltration in old neurogenic niches. Nature. (2019) 571:205–10. doi: 10.1038/s41586-019-1362-5, PMID: 31270459 PMC7111535

[68] Faust HJ Zhang H Han J Wolf MT Jeon OH Sadtler K. IL-17 and immunologically induced senescence regulate response to injury in osteoarthritis. J Clin Invest. (2020) 130:5493–507. doi: 10.1172/JCI134091, PMID: 32955487 PMC7524483

[69] Larochelle C Wasser B Jamann H Löffel JT Cui QL Tastet O. Pro-inflammatory T helper 17 directly harms oligodendrocytes in neuroinflammation. Proc Natl Acad Sci U S A. (2021) 118:e2025813118. doi: 10.1073/pnas.2025813118, PMID: 34417310 PMC8403833

[70] Voskuhl R Itoh Y. The X factor in neurodegeneration. J Exp Med. (2022) 219:e20211488. doi: 10.1084/jem.20211488, PMID: 36331399 PMC9641640

[71] Rawji KS Kappen J Tang W Teo W Plemel JR Stys PK. Deficient surveillance and phagocytic activity of myeloid cells within demyelinated lesions in aging mice visualized by ex vivo live multiphoton imaging. Rapid Commun. (2018) 38:1973–88. doi: 10.1523/JNEUROSCI.2341-17.2018, PMID: 29363580 PMC6705888

[72] Neumann B Segel M Chalut KJ Franklin RJ. Remyelination and ageing: reversing the ravages of time. Mult Scler J. (2019) 25:1835–41. doi: 10.1177/1352458519884006, PMID: 31687878 PMC7682531

[73] Palmer AL Ousman SS. Astrocytes and aging. Front Aging Neurosci. (2018) 10:337. doi: 10.3389/fnagi.2018.00337 30416441 PMC6212515

[74] Nicaise AM Wagstaff LJ Willis CM Paisie C Chandok H Robson P. Cellular senescence in progenitor cells contributes to diminished remyelination potential in progressive multiple sclerosis. Proc Natl Acad Sci U S A. (2019) 116:9030–9. doi: 10.1073/pnas.1818348116, PMID: 30910981 PMC6500153

[75] Habib R Ocklenburg S Hoffjan S Haghikia A Epplen JT Arning L. Association between shorter leukocyte telomeres and multiple sclerosis. J Neuroimmunol. (2020) 341:577187. doi: 10.1016/j.jneuroim.2020.577187, PMID: 32050150

[76] Krysko KM Henry RG Cree BAC Lin J University of California, San Francisco MS‐EPIC Team Caillier S. Telomere length is associated with disability progression in multiple sclerosis. Ann Neurol. (2019) 86:671–82. doi: 10.1002/ana.25592, PMID: 31486104 PMC7135931

[77] Zuroff L Rezk A Shinoda K Espinoza DA Elyahu Y Zhang B. Immune aging in multiple sclerosis is characterized by abnormal CD4 T cell activation and increased frequencies of cytotoxic CD4 T cells with advancing age. EBioMedicine. (2022) 82:104179. doi: 10.1016/j.ebiom.2022.104179, PMID: 35868128 PMC9305354

[78] Eschborn M Pawlitzki M Wirth T Nelke C Pfeuffer S Schulte-Mecklenbeck A. Evaluation of age-dependent immune signatures in patients with multiple sclerosis. Neurology. (2021) 8:e1094. doi: 10.1212/NXI.0000000000001094, PMID: 34667129 PMC8529419

[79] Pender MP Csurhes PA Pfluger CMM Burrows SR. CD8 T cell deficiency impairs control of Epstein--Barr virus and worsens with age in multiple sclerosis. J Neurol Neurosurg Psychiatry. (2012) 83:353–4. doi: 10.1136/jnnp-2011-300213, PMID: 21791511 PMC3277686

[80] Ruan P Wang S Yang M Wu H. The ABC-associated immunosenescence and lifestyle interventions in autoimmune disease. Rheumatol Immunol Res. (2022) 3:128–35. doi: 10.2478/rir-2022-0021, PMID: 36788975 PMC9895871

[81] Sachinidis A Garyfallos A. Involvement of age-associated B cells in EBV-triggered autoimmunity. Immunol Res. (2022) 70:546–9. doi: 10.1007/s12026-022-09291-y, PMID: 35575824 PMC9109436

[82] Valdes AM Andrew T Gardner JP Kimura M Oelsner E Cherkas LF. Obesity, cigarette smoking, and telomere length in women. Lancet. (2005) 366:662–4. doi: 10.1016/S0140-6736(05)66630-5, PMID: 16112303

[83] Versini M Jeandel P-Y Rosenthal E Shoenfeld Y. Obesity in autoimmune diseases: not a passive bystander. Autoimmun Rev. (2014) 13:981–1000. doi: 10.1016/j.autrev.2014.07.001, PMID: 25092612

[84] Yang H Youm Y-H Vandanmagsar B Rood J Kumar KG Butler AA. Obesity accelerates thymic aging. Blood. (2009) 114:3803–12. doi: 10.1182/blood-2009-03-213595, PMID: 19721009 PMC2773495

[85] Taylor JM Li A McLachlan CS. Immune cell profile and immune-related gene expression of obese peripheral blood and liver tissue. FEBS Lett. (2022) 596:199–210. doi: 10.1002/1873-3468.14248, PMID: 34850389

[86] Prattichizzo F de Nigris V Spiga R Mancuso E la Sala L Antonicelli R. Inflammageing and metaflammation: the yin and yang of type 2 diabetes. Ageing Res Rev. (2018) 41:1–17. doi: 10.1016/j.arr.2017.10.003, PMID: 29081381

[87] Demnitz N Stathi A Withall J Stainer C Seager P de Koning J. Hippocampal maintenance after a 12-month physical activity intervention in older adults: the REACT MRI study. NeuroImage Clin. (2022) 35:102762. doi: 10.1016/j.nicl.2021.102762, PMID: 35361556 PMC9421470

[88] Riemenschneider M Hvid LG Ringgaard S Nygaard MKE Eskildsen SF Gaemelke T. Investigating the potential disease-modifying and neuroprotective efficacy of exercise therapy early in the disease course of multiple sclerosis: the early multiple sclerosis exercise study (EMSES). Mult Scler J. (2022) 28:1620–9. doi: 10.1177/13524585221079200, PMID: 35296183

[89] Lozinski BM de Almeida LGN Silva C Dong Y Brown D Chopra S. Exercise rapidly alters proteomes in mice following spinal cord demyelination. Sci Rep. (2021) 11:7239. doi: 10.1038/s41598-021-86593-5, PMID: 33790323 PMC8012633

[90] Fitzgerald KC Bhargava P Smith MD Vizthum D Henry-Barron B Kornberg MD. Intermittent calorie restriction alters T cell subsets and metabolic markers in people with multiple sclerosis. EBioMedicine. (2022) 82:104124. doi: 10.1016/j.ebiom.2022.104124, PMID: 35816900 PMC9283513

[91] Negrotto L Farez MF Correale J. Immunologic effects of metformin and pioglitazone treatment on metabolic syndrome and multiple sclerosis. JAMA Neurol. (2016) 73:520–8. doi: 10.1001/jamaneurol.2015.4807, PMID: 26953870

[92] Roy DG Chen J Mamane V Ma EH Muhire BM Sheldon RD. Methionine metabolism shapes T helper cell responses through regulation of epigenetic reprogramming. Cell Metab. (2020) 31:250–266.e9. doi: 10.1016/j.cmet.2020.01.006, PMID: 32023446

[93] Conway J Duggal NA. Ageing of the gut microbiome: potential influences on immune senescence and inflammageing. Ageing Res Rev. (2021) 68:101323. doi: 10.1016/j.arr.2021.101323, PMID: 33771720

[94] iMSMS Consortium. Gut microbiome of multiple sclerosis patients and paired household healthy controls reveal associations with disease risk and course. Cells. (2022) 185:3467–3486.e16. doi: 10.1016/j.cell.2022.08.021, PMID: 36113426 PMC10143502

[95] Conway DS Thompson NR Cohen JA. Influence of hypertension, diabetes, hyperlipidemia, and obstructive lung disease on multiple sclerosis disease course. Mult Scler. (2017) 23:277–85. doi: 10.1177/1352458516650512, PMID: 27230791

[96] Nagai M Hoshide S Ishikawa J Shimada K Kario K. Ambulatory blood pressure as an independent determinant of brain atrophy and cognitive function in elderly hypertension. J Hypertens. (2008) 26:1636–41. doi: 10.1097/HJH.0b013e3283018333, PMID: 18622243

[97] Dai D Sharma A Phillips A Lobo C. Patterns of comorbidity and multimorbidity among patients with multiple sclerosis in a large US commercially insured and Medicare advantage population. J Heal Econ Outcomes Res. (2022) 9:125–33. doi: 10.36469/jheor.2022.38669 PMC9684016 36475279

[98] Graves JS Krysko KM Hua LH Absinta M Franklin RJM Segal BM. Ageing and multiple sclerosis. Lancet Neurol. (2022) 4422:1–12. doi: 10.1016/s1474-4422(22)00184-3 36216015

[99] Palladino R Marrie RA Majeed A Chataway J. Evaluating the risk of macrovascular events and mortality among people with multiple sclerosis in England. JAMA Neurol. (2020) 77:820–8. doi: 10.1001/jamaneurol.2020.0664, PMID: 32364569 PMC7199174

[100] Marrie RA Garland A Schaffer SA Fransoo R Leung S Yogendran M. Traditional risk factors may not explain increased incidence of myocardial infarction in MS. Neurology. (2019) 92:e1624–33. doi: 10.1212/WNL.0000000000007251, PMID: 30842298

[101] Marrie RA Fisk J Tremlett H Wolfson C Warren S Blanchard J. Differing trends in the incidence of vascular comorbidity in MS and the general population. Neurol Clin Pract. (2016) 6:120–8. doi: 10.1212/CPJ.0000000000000230, PMID: 27104065 PMC4828682

[102] Low ASL Symmons DPM Lunt M Mercer LK Gale CP Watson KD. Relationship between exposure to tumour necrosis factor inhibitor therapy and incidence and severity of myocardial infarction in patients with rheumatoid arthritis. Ann Rheum Dis. (2017) 76:654–60. doi: 10.1136/annrheumdis-2016-209784, PMID: 28073800 PMC5530342

[103] Wang B Li X Li H Xiao L Zhou Z Chen K. Clinical, radiological and pathological characteristics between cerebral small vessel disease and multiple sclerosis: a review. Front Neurol. (2022) 13:1–11. doi: 10.3389/fneur.2022.841521, PMID: 35812110 PMC9263123

[104] Azevedo CJ Cen SY Jaberzadeh A Zheng L Hauser SL Pelletier D. Contribution of normal aging to brain atrophy in MS. Neurol Neuroimmunol Neuroinflamm. (2019) 6:1–10. doi: 10.1212/NXI.0000000000000616 PMC6807662 32330116

[105] Hearn AP Silber E. Osteoporosis in multiple sclerosis. Mult Scler. (2010) 16:1031–43. doi: 10.1177/1352458510368985, PMID: 20551086

[106] Lynch S Baker S Nashatizadeh M Thuringer A Thelen J Bruce J. Disability measurement in multiple sclerosis patients 55 years and older: what is the expanded disability status scale really telling clinicians? Mult Scler Relat Disord. (2020) 2021:102724. doi: 10.1016/j.msard.2020.102724 33609959

[107] Tremblay A Kim C Estefania B Elaine R Pierre D Isabelle R. The effects of aging and disease duration on cognition in multiple sclerosis. Brain Cogn. (2020) 146:105650. doi: 10.1016/j.bandc.2020.105650 33212390

[108] O’Brien JT Thomas A. Vascular dementia. Lancet. (2015) 386:1698–706. doi: 10.1016/S0140-6736(15)00463-8, PMID: 26595643

[109] Mahmoudi E Sadaghiyani S Lin P Kamdar N Norcott A Peterson MD. Diagnosis of Alzheimer's disease and related dementia among people with multiple sclerosis: large cohort study, USA. MSARD. (2022) 57:103351. doi: 10.1016/j.msard.2021.103351, PMID: 35158460

[110] Luczynski P Laule C Hsiung GYR Moore GRW Tremlett H. Coexistence of multiple sclerosis and Alzheimer’s disease: a review. Mult Scler Relat Disord. (2018) 2019:232–8. doi: 10.1016/j.msard.2018.10.109 30415025

[111] Benedict RHB Amato MP DeLuca J Geurts JJG. Cognitive impairment in multiple sclerosis: clinical management, MRI, and therapeutic avenues. Lancet Neurol. (2020) 19:860–71. doi: 10.1016/S1474-4422(20)30277-5, PMID: 32949546 PMC10011205

[112] Roth A Denney D Burns J Lynch S. Cognition in older patients with multiple sclerosis compared to patients with amnestic mild cognitive impairment and healthy older adults. Neuropsychology. (2018) 32:654–63. doi: 10.1037/neu0000453, PMID: 29939057 PMC6126957

[113] Fried LP Tangen CM Walston J Newman AB Hirsch C Gottdiener J. Frailty in older adults: evidence for a phenotype. J Gerontol A Biol Sci Med Sci. (2001) 56:M146–57. doi: 10.1093/gerona/56.3.M146, PMID: 11253156

[114] Ayrignac X Larochelle C Keezer M Roger E Poirier J Lahav B. Frailty in ageing persons with multiple sclerosis. Mult Scler J. (2021) 27:613–20. doi: 10.1177/1352458520923945, PMID: 32458728

[115] Jakimovski D Eckert SP Zivadinov R Weinstock-Guttman B. Considering patient age when treating multiple sclerosis across the adult lifespan. Expert Rev Neurother. (2021) 21:353–64. doi: 10.1080/14737175.2021.1886082, PMID: 33595379

[116] Vollmer BL Wolf AB Sillau S Corboy JR Alvarez E. Evolution of disease modifying therapy benefits and risks: an argument for De-escalation as a treatment paradigm for patients with multiple sclerosis. Front Neurol. (2022) 12:1–8. doi: 10.3389/fneur.2021.799138 PMC8821102 35145470

[117] Ng HS Graf J Zhu F Kingwell E Aktas O Albrecht P. Disease-modifying drug uptake and health service use in the ageing MS population. Front Immunol. (2022) 12:1–11. doi: 10.3389/fimmu.2021.794075, PMID: 35095869 PMC8792855

[118] Miller AE O’Connor P Wolinsky JS Confavreux C Kappos L Olsson TP. Pre-specified subgroup analyses of a placebo-controlled phase III trial (TEMSO) of oral teriflunomide in relapsing multiple sclerosis. Mult Scler J. (2012) 18:1625–32. doi: 10.1177/1352458512450354, PMID: 22723573 PMC3573676

[119] Gold R Kappos L Arnold DL Bar-Or A Giovannoni G Selmaj K. Placebo-controlled phase 3 study of oral BG-12 for relapsing multiple sclerosis. N Engl J Med. (2012) 367:1098–107. doi: 10.1056/NEJMoa1114287, PMID: 22992073

[120] Fox RJ Miller DH Phillips JT Hutchinson M Havrdova E Kita M. Placebo-controlled phase 3 study of oral BG-12 or glatiramer in multiple sclerosis. N Engl J Med. (2012) 367:1087–97. doi: 10.1056/NEJMoa1206328, PMID: 22992072

[121] Devonshire V Havrdova E Radue EW O'Connor P Zhang-Auberson L Agoropoulou C. Relapse and disability outcomes in patients with multiple sclerosis treated with fingolimod: subgroup analyses of the double-blind, randomised, placebo-controlled FREEDOMS study. Lancet Neurol. (2012) 11:420–8. doi: 10.1016/S1474-4422(12)70056-X, PMID: 22494956

[122] Kappos L Bar-Or A Cree BAC Fox RJ Giovannoni G Gold R. Siponimod versus placebo in secondary progressive multiple sclerosis (EXPAND): a double-blind, randomised, phase 3 study. Lancet. (2018) 391:1263–73. doi: 10.1016/S0140-6736(18)30475-6, PMID: 29576505

[123] Comi G Kappos L Selmaj KW Bar-Or A Arnold DL Steinman L. Safety and efficacy of ozanimod versus interferon beta-1a in relapsing multiple sclerosis (SUNBEAM): a multicentre, randomised, minimum 12-month, phase 3 trial. Lancet Neurol. (2019) 18:1009–20. doi: 10.1016/S1474-4422(19)30239-X, PMID: 31492651

[124] Cohen JA Comi G Selmaj KW Bar-Or A Arnold DL Steinman L. Safety and efficacy of ozanimod versus interferon beta-1a in relapsing multiple sclerosis (RADIANCE): a multicentre, randomised, 24-month, phase 3 trial. Lancet Neurol. (2019) 18:1021–33. doi: 10.1016/S1474-4422(19)30238-8, PMID: 31492652

[125] Giovannoni G Cook S Rammohan K Rieckmann P Sørensen PS Vermersch P. Sustained disease-activity-free status in patients with relapsing-remitting multiple sclerosis treated with cladribine tablets in the CLARITY study: a post-hoc and subgroup analysis. Lancet Neurol. (2011) 10:329–37. doi: 10.1016/S1474-4422(11)70023-0, PMID: 21397565

[126] Turner B Cree BAC Kappos L Montalban X Papeix C Wolinsky JS. Ocrelizumab efficacy in subgroups of patients with relapsing multiple sclerosis. J Neurol. (2019) 266:1182–93. doi: 10.1007/s00415-019-09248-6, PMID: 30820738 PMC6469695

[127] Montalban X Hauser SL Kappos L Arnold DL Bar-Or A Comi G. Ocrelizumab versus placebo in primary progressive multiple sclerosis. N Engl J Med. (2017) 376:209–20. doi: 10.1056/NEJMoa1606468, PMID: 28002688

[128] Hauser SL Bar-Or A Cohen JA Comi G Correale J Coyle PK. Ofatumumab versus Teriflunomide in Multiple Sclerosis. N Engl J Med. (2020) 383:546–57. doi: 10.1056/NEJMoa1917246, PMID: 32757523

[129] for the AFFIRM and SENTINEL Investigators Hutchinson M Kappos L Calabresi PA Confavreux C Giovannoni G. The efficacy of natalizumab in patients with relapsing multiple sclerosis: subgroup analyses of AFFIRM and SENTINEL. J Neurol. (2009) 256:405–15. doi: 10.1007/s00415-009-0093-1, PMID: 19308305

[130] Zhang Y Caldito NG Shirani A Salter A Cutter G Culpepper W. Aging and efficacy of disease-modifying therapies in multiple sclerosis: a meta- analysis of clinical trials Yinan. Ther Adv Neurol Disord. (2020) 13:1–10. doi: 10.1177/https PMC7838219 33552235

[131] Comi G Bermel R Bar-Or A McGinley M Arnold D Henry R. A multicentre, open label, single-arm, phase 3b study (CONSONANCE) to assess the effectiveness and safety of ocrelizumab in patients with primary and secondary progressive multiple sclerosis: year-1 interim analysis (P1-1.Virtual). Neurology. (2022) 98:652.

[132] Buscarinu MC Reniè R Morena E Romano C Bellucci G Marrone A. Late-onset MS: disease course and safety-efficacy of DMTS. Front Neurol. (2022) 13:1–7. doi: 10.3389/fneur.2022.829331, PMID: 35356454 PMC8960027

[133] Vaughn CB Jakimovski D Kavak KS Ramanathan M Benedict RHB Zivadinov R. Epidemiology and treatment of multiple sclerosis in elderly populations. Nat Rev Neurol. (2019) 15:329–42. doi: 10.1038/s41582-019-0183-3, PMID: 31000816

[134] Ostolaza A Corroza J Ayuso T. Multiple sclerosis and aging: comorbidity and treatment challenges. Mult Scler Relat Disord. (2021) 2021:102815. doi: 10.1016/j.msard.2021.102815 33581613

[135] Trojano M Tintore M Montalban X Hillert J Kalincik T Iaffaldano P. Treatment decisions in multiple sclerosis - insights from real-world observational studies. Nat Rev Neurol. (2017) 13:105–18. doi: 10.1038/nrneurol.2016.188, PMID: 28084327

[136] Grebenciucova E Berger JR. Immunosenescence: the role of aging in the predisposition to neuro-infectious complications arising from the treatment of multiple sclerosis. Curr Neurol Neurosci Rep. (2017) 17:61. doi: 10.1007/s11910-017-0771-9 28669032

[137] Prosperini L de Rossi N Scarpazza C Moiola L Cosottini M Gerevini S. Natalizumab-related progressive multifocal leukoencephalopathy in multiple sclerosis: findings from an Italian independent registry. PLoS One. (2016) 11:e0168376. doi: 10.1371/journal.pone.0168376, PMID: 27997580 PMC5172579

[138] Luna G Alping P Burman J Fink K Fogdell-Hahn A Gunnarsson M. Infection risks among patients with multiple sclerosis treated with fingolimod, natalizumab, rituximab, and injectable therapies. JAMA Neurol. (2020) 77:184–91. doi: 10.1001/jamaneurol.2019.3365, PMID: 31589278 PMC6784753

[139] Barmettler S Ong M Farmer JR Choi H Walter J. Association of immunoglobulin levels, infectious risk, and mortality with rituximab and hypogammaglobulinemia. JAMA Netw Open. (2018) 1:1–14. doi: 10.1001/jamanetworkopen.2018.4169 PMC6324375 30646343

[140] Sormani MP de Rossi N Schiavetti I Carmisciano L Cordioli C Moiola L. Disease-modifying therapies and coronavirus disease 2019 severity in multiple sclerosis. Ann Neurol. (2021) 89:780–9. doi: 10.1002/ana.26028, PMID: 33480077 PMC8013440

[141] Simpson-Yap S de Brouwer E Kalincik T Rijke N Hillert JA Walton C. Associations of disease-modifying therapies with COVID-19 severity in multiple sclerosis. Neurology. (2021) 97:e1870–85. doi: 10.1212/WNL.0000000000012753, PMID: 34610987 PMC8601210

[142] Reder AT Centonze D Naylor ML Nagpal A Rajbhandari R Altincatal A. COVID-19 in patients with multiple sclerosis: associations with disease-modifying therapies. CNS Drugs. (2021) 35:317–30. doi: 10.1007/s40263-021-00804-1, PMID: 33743151 PMC7980129

[143] Louapre C Collongues N Stankoff B Giannesini C Papeix C Bensa C. Clinical characteristics and outcomes in patients with coronavirus disease 2019 and multiple sclerosis. JAMA Neurol. (2020) 77:1079–88. doi: 10.1001/jamaneurol.2020.2581, PMID: 32589189 PMC7320356

[144] Tallantyre EC Vickaryous N Anderson V Asardag AN Baker D Bestwick J. COVID-19 vaccine response in people with multiple sclerosis. Ann Neurol. (2022) 91:89–100. doi: 10.1002/ana.26251, PMID: 34687063 PMC8652739

[145] Ritter C Svačina MKR Bobylev I Joshi A Schneider T Lehmann HC. Impact of age and polytherapy on fingolimod induced bradycardia: a preclinical study. J Neuroimmune Pharmacol Off J Soc NeuroImmune Pharmacol. (2017) 12:204–9. doi: 10.1007/s11481-017-9727-8, PMID: 28150133

[146] Ghajarzadeh M Mohammadi A Sahraian MA. Risk of cancer in multiple sclerosis (MS): A systematic review and meta-analysis. Autoimmun Rev. (2020) 19:102650. doi: 10.1016/j.autrev.2020.102650, PMID: 32801049

[147] Nielsen NM Rostgaard K Rasmussen S Koch-Henriksen N Storm HH Melbye M. Cancer risk among patients with multiple sclerosis: a population-based register study. Int J Cancer. (2006) 118:979–84. doi: 10.1002/ijc.21437, PMID: 16152598

[148] Lebrun C Rocher F. Cancer risk in patients with multiple sclerosis: potential impact of disease – modifying drugs. CNS Drugs. (2018) 32:939–49. doi: 10.1007/s40263-018-0564-y, PMID: 30143945

[149] Lebrun C Vermersch P Brassat D Defer G Rumbach L Clavelou P. Cancer and multiple sclerosis in the era of disease-modifying treatments. J Neurol. (2011) 258:1304–11. doi: 10.1007/s00415-011-5929-9, PMID: 21293872

[150] Kingwell E Evans C Zhu F Oger J Hashimoto S Tremlett H. Assessment of cancer risk with β-interferon treatment for multiple sclerosis. J Neurol Neurosurg Psychiatry. (2014) 85:1096–102. doi: 10.1136/jnnp-2013-307238, PMID: 24594506

[151] Hales CM Servais J Martin CB Kohen D. Prescription drug use among adults aged 40-79 in the United States and Canada. NCHS Data Brief. (2019) 347:1–8. 31442200

[152] Frahm N Hecker M Zettl UK. Polypharmacy among patients with multiple sclerosis: a qualitative systematic review. Expert Opin Drug Saf. (2020) 19:139–45. doi: 10.1080/14740338.2020.1720646, PMID: 31965869

[153] Organization WH. Medication safety in polypharmacy: Technical report World Health Organization (2019) 61.

[154] Dechanont S Maphanta S Butthum B Kongkaew C. Hospital admissions/visits associated with drug-drug interactions: a systematic review and meta-analysis. Pharmacoepidemiol Drug Saf. (2014) 23:489–97. doi: 10.1002/pds.3592, PMID: 24616171

[155] Chertcoff A Ng HS Zhu F Zhao Y Tremlett H. Polypharmacy and multiple sclerosis: a population-based study. Mult Scler J. (2023) 29:107–18. doi: 10.1177/13524585221122207, PMID: 36301629 PMC9896267

[156] Hartung DM Johnston KA McGregor JC Bourdette DN. Characteristics of prescription drug use among individuals with multiple sclerosis in the US Medicare population. Int J MS Care. (2022) 24:91–7. doi: 10.7224/1537-2073.2021-062 PMC9017658 35462869

[157] Cameron MH Karstens L Hoang P Bourdette D Lord S. Medications are associated with falls in people with multiple sclerosis. Int J MS Care. (2015) 17:207–14. doi: 10.7224/1537-2073.2014-076, PMID: 26472941 PMC4599357

[158] Thelen JM Lynch SG Bruce AS Hancock LM Bruce JM. Polypharmacy in multiple sclerosis: relationship with fatigue, perceived cognition, and objective cognitive performance. J Psychosom Res. (2014) 76:400–4. doi: 10.1016/j.jpsychores.2014.02.013, PMID: 24745782

[159] Debus JL Bachmann P Frahm N Mashhadiakbar P Langhorst SE. Associated factors of potential drug-drug interactions and drug–food interactions in patients with multiple sclerosis. Ther Adv Chronic Dis. (2022) 13:1–19. doi: 10.1177/https PMC9358348 35959503

[160] Bourdette D Herink M. Polypharmacy in multiple sclerosis: more is not necessarily better. Mult Scler J. (2023) 29:1–5. doi: 10.1177/https 36239156

[161] Foley JF Defer G Ryerson LZ Cohen JA Arnold DL Butzkueven H. Comparison of switching to 6-week dosing of natalizumab versus continuing with 4-week dosing in patients with relapsing-remitting multiple sclerosis (NOVA): a randomised, controlled, open-label, phase 3b trial. Lancet Neurol. (2022) 21:608–19. doi: 10.1016/S1474-4422(22)00143-0, PMID: 35483387

[162] Rolfes L Pawlitzki M Pfeuffer S Nelke C Lux A Pul R. Ocrelizumab extended interval dosing in multiple sclerosis in times of COVID-19. Neurology. (2021) 8:e1035. doi: 10.1212/NXI.0000000000001035, PMID: 34261812 PMC8362352

[163] Hua LH Harris H Conway D Thompson NR. Changes in patient-reported outcomes between continuers and discontinuers of disease modifying therapy in patients with multiple sclerosis over age 60. Mult Scler Relat Disord. (2018) 2019:252–6. doi: 10.1016/j.msard.2019.02.028 30851638

[164] McGinley MP Cola PA Fox RJ Cohen JA Corboy JR Miller DM. Perspectives of individuals with multiple sclerosis on discontinuation of disease-modifying therapies. Mult Scler J. (2020) 26:1581–9. doi: 10.1177/1352458519867314, PMID: 31368401

[165] Kister I Spelman T Patti F Duquette P Trojano M Izquierdo G. Predictors of relapse and disability progression in MS patients who discontinue disease-modifying therapy. J Neurol Sci. (2018) 391:72–6. doi: 10.1016/j.jns.2018.06.001, PMID: 30103975

[166] Yano H Gonzalez C Healy BC Glanz BI Weiner HL Chitnis T. Discontinuation of disease-modifying therapy for patients with relapsing-remitting multiple sclerosis: effect on clinical and MRI outcomes. Mult Scler Relat Disord. (2019) 35:119–27. doi: 10.1016/j.msard.2019.07.021 31374460

[167] Bsteh G Feige J Ehling R Auer M Hegen H di Pauli F. Discontinuation of disease-modifying therapies in multiple sclerosis – clinical outcome and prognostic factors. Mult Scler. (2017) 23:1241–8. doi: 10.1177/1352458516675751, PMID: 27765877

[168] Bsteh G Hegen H Riedl K Altmann P Auer M Berek K. Quantifying the risk of disease reactivation after interferon and glatiramer acetate discontinuation in multiple sclerosis: the VIAADISC score. Eur J Neurol. (2021) 28:1609–16. doi: 10.1111/ene.14705, PMID: 33370478 PMC8248019

[169] Kaminsky AL Omorou AY Soudant M Pittion-Vouyovitch S Michaud M Anxionnat R. Discontinuation of disease-modifying treatments for multiple sclerosis in patients aged over 50 with disease inactivity. J Neurol. (2020) 267:3518–27. doi: 10.1007/s00415-020-10029-9, PMID: 32617659

[170] Jakimovski D Kavak KS Vaughn CB Goodman AD Coyle PK Krupp L. Discontinuation of disease modifying therapies is associated with disability progression regardless of prior stable disease and age. Mult Scler Relat Disord. (2022) 57:103406. doi: 10.1016/j.msard.2021.103406, PMID: 34915316

[171] Chappuis M Rousseau C Bajeux E Wiertlewski S Laplaud D le Page E. Discontinuation of second- versus first-line disease-modifying treatment in middle-aged patients with multiple sclerosis. J Neurol. (2023) 270:413–22. doi: 10.1007/s00415-022-11341-2, PMID: 36121558

[172] Fagius J Feresiadou A Larsson EM Burman J. Discontinuation of disease modifying treatments in middle aged multiple sclerosis patients. First line drugs vs natalizumab. Mult Scler Relat Disord. (2017) 12:82–7. doi: 10.1016/j.msard.2017.01.009 28283113

[173] Pantazou V Pot C Du Pasquier R Le Goff G Théaudin M. Recurrence of disease activity after fingolimod discontinuation in older patients previously stable on treatment. Mult Scler Relat Disord. (2021) 51:102918. doi: 10.1016/j.msard.2021.102918 33838521

[174] Juto A Fink K Al Nimer F Piehl F. Interrupting rituximab treatment in relapsing-remitting multiple sclerosis; no evidence of rebound disease activity. Mult Scler Relat Disord. (2019) 2020:101468. doi: 10.1016/j.msard.2019.101468 31683231

[175] Conway BL Zeydan B Uygunoğlu U Novotna M Siva A Pittock SJ. Age is a critical determinant in recovery from multiple sclerosis relapses. Mult Scler J. (2019) 25:1754–63. doi: 10.1177/1352458518800815, PMID: 30303037

[176] Sorensen PS Giovannoni G Montalban X Thalheim C Zaratin P Comi G. The multiple sclerosis care unit. Mult Scler J. (2019) 25:627–36. doi: 10.1177/1352458518807082, PMID: 30351211 PMC6439947

[177] Marrie RA Donkers SJ Jichici D Hrebicek O Metz L. Models of Care in Multiple Sclerosis: A survey of Canadian health providers. Front Neurol. (2022) 13:1–12. doi: 10.3389/fneur.2022.904757 PMC9163821 35669877

[178] Hartung HP Meuth SG Miller DM Comi G. Stopping disease-modifying therapy in relapsing and progressive multiple sclerosis. Curr Opin Neurol. (2021) 34:598–603. doi: 10.1097/WCO.0000000000000960, PMID: 33990101

[179] Gross RH Corboy JR. Monitoring, switching, and stopping multiple sclerosis disease-modifying therapies. Continuum (Minneap Minn). (2019) 25:715–35. doi: 10.1212/CON.0000000000000738, PMID: 31162313

[180] Freedman MS Devonshire V Duquette P Giacomini PS Giuliani F Levin MC. Treatment optimization in multiple sclerosis: Canadian MS working group recommendations. Canad J Neurol Sci. (2020) 47:437–55. doi: 10.1017/cjn.2020.66, PMID: 32654681

